# The international and intercontinental spread and expansion of antimicrobial-resistant *Salmonella* Typhi: a genomic epidemiology study

**DOI:** 10.1016/S2666-5247(22)00093-3

**Published:** 2022-08

**Authors:** Kesia Esther da Silva, Arif Mohammad Tanmoy, Agila Kumari Pragasam, Junaid Iqbal, Mohammad Saiful Islam Sajib, Ankur Mutreja, Balaji Veeraraghavan, Dipesh Tamrakar, Farah Naz Qamar, Gordon Dougan, Isaac Bogoch, Jessica C Seidman, Jivan Shakya, Krista Vaidya, Megan E Carey, Rajeev Shrestha, Seema Irfan, Stephen Baker, Steve P Luby, Yanjia Cao, Zoe Anne Dyson, Denise O Garrett, Jacob John, Gagandeep Kang, Yogesh Hooda, Samir K Saha, Senjuti Saha, Jason R Andrews

**Affiliations:** a Division of Infectious Diseases and Geographic Medicine, Stanford University, Stanford, CA, USA; b Child Health Research Foundation, Dhaka, Bangladesh; c Department of Medical Microbiology and Infectious Diseases, Erasmus University Medical Center, Rotterdam, Netherlands; d Department of Clinical Microbiology, Christian Medical College, Vellore, India; e Department of Community Health, Christian Medical College, Vellore, India; f The Wellcome Trust Research Laboratory, Division of Gastrointestinal Sciences, Christian Medical College, Vellore, India; g Department of Pediatrics and Child Health, Aga Khan University, Karachi, Pakistan; h Department of Pathology and Microbiology, Aga Khan University, Karachi, Pakistan; i Institute of Biodiversity, Animal Health and Comparative Medicine, University of Glasgow, Glasgow, UK; j Cambridge Institute of Therapeutic Immunology & Infectious Disease, Department of Medicine, University of Cambridge, Cambridge, UK; k Department of Community Medicine, Dhulikhel Hospital, Kathmandu University Hospital, Kathmandu, Nepal; l Department of Pharmacology, Dhulikhel Hospital, Kathmandu University Hospital, Kathmandu, Nepal; m Research and Development Division, Dhulikhel Hospital, Kathmandu University Hospital, Kathmandu, Nepal; n Department of Medicine, Cambridge Biomedical Campus, Cambridge, UK; o University of Cambridge School of Clinical Medicine, Cambridge Biomedical Campus, Cambridge, UK; p Department of Medicine, University of Toronto, Toronto, ON, Canada; q Applied Epidemiology, Sabin Vaccine Institute, Washington, DC, USA; r Central Department of Microbiology, Tribhuvan University, Kirtipur, Nepal; s Department of Geography, The University of Hong Kong, Hong Kong Special Administrative Region, China; t Department of Infection Biology, Faculty of Infectious and Tropical Diseases, London School of Hygiene & Tropical Medicine, London, UK; u Department of Infectious Diseases, Central Clinical School, Monash University, Melbourne, VIC, Australia; v Wellcome Sanger Institute, Wellcome Genome Campus, Cambridge, UK; w MRC Laboratory Molecular Biology, Cambridge, UK; x Department of Microbiology, Dhaka Shishu Hospital, Dhaka, Bangladesh

## Abstract

**Background:**

The emergence of increasingly antimicrobial-resistant *Salmonella enterica* serovar Typhi (*S* Typhi) threatens to undermine effective treatment and control. Understanding where antimicrobial resistance in *S* Typhi is emerging and spreading is crucial towards formulating effective control strategies.

**Methods:**

In this genomic epidemiology study, we sequenced the genomes of 3489 *S* Typhi strains isolated from prospective enteric fever surveillance studies in Nepal, Bangladesh, Pakistan, and India (between 2014 and 2019), and combined these with a global collection of 4169 *S* Typhi genome sequences isolated between 1905 and 2018 to investigate the temporal and geographical patterns of emergence and spread of antimicrobial-resistant *S* Typhi. We performed non-parametric phylodynamic analyses to characterise changes in the effective population size of fluoroquinolone-resistant, extensively drug-resistant (XDR), and azithromycin-resistant *S* Typhi over time. We inferred timed phylogenies for the major *S* Typhi sublineages and used ancestral state reconstruction methods to estimate the frequency and timing of international and intercontinental transfers.

**Findings:**

Our analysis revealed a declining trend of multidrug resistant typhoid in south Asia, except for Pakistan, where XDR *S* Typhi emerged in 2016 and rapidly replaced less-resistant strains. Mutations in the quinolone-resistance determining region (QRDR) of *S* Typhi have independently arisen and propagated on at least 94 occasions, nearly all occurring in south Asia. Strains with multiple QRDR mutations, including triple mutants with high-level fluoroquinolone resistance, have been increasing in frequency and displacing strains with fewer mutations. Strains containing *acrB* mutations, conferring azithromycin resistance, emerged in Bangladesh around 2013 and effective population size of these strains has been steadily increasing. We found evidence of frequent international (n=138) and intercontinental transfers (n=59) of antimicrobial-resistant *S* Typhi, followed by local expansion and replacement of drug-susceptible clades.

**Interpretation:**

Independent acquisition of plasmids and homoplastic mutations conferring antimicrobial resistance have occurred repeatedly in multiple lineages of *S* Typhi, predominantly arising in south Asia before spreading to other regions.

**Funding:**

Bill & Melinda Gates Foundation.

## Introduction

Typhoid fever, the disease caused by *Salmonella enterica* serovar Typhi (*S* Typhi), remains a major public health concern worldwide,^1^ causing 11 million cases and more than 100 000 deaths annually.^2^, ^3^ The highest incidence rates occur in south Asia, which contains 70% of the global disease burden, but substantial morbidity and mortality also occur in sub-Saharan Africa, southeast Asia, and Oceania.^4^

The effectiveness of antimicrobial therapy has been threatened by the emergence and expansion of antimicrobial-resistant strains. Multidrug-resistant variants, harbouring genes encoding resistance to ampicillin, chloramphenicol, and trimethoprim–sulfamethoxazole, first emerged in the 1970s; subsequently, a single lineage (4.3.1) associated with multidrug-resistance among the H58 haplotype became globally dominant.^5^, ^6^ Fluoroquinolones were initially effective against multidrug-resistant *S* Typhi and became the mainstay of therapy in the 1990s. However, by the 2010s, the majority of *S* Typhi in south Asia contained mutations in the quinolone resistance-determining regions (QRDR).^7^, ^8^ In 2016, a large outbreak of *S* Typhi containing plasmid-mediated resistance to third generation cephalosporins and fluoroquinolone, and chromosomally located genes encoding multidrug-resistance were identified in Pakistan and termed extensively drug-resistant (XDR).^9^ In 2021 a single polymorphism in the AcrB efflux pump conferring resistance to azithromycin was found to have independently arisen in multiple lineages of *S* Typhi, threatening the efficacy of all oral antimicrobials for typhoid treatment.^10^

**Research in context**

**Evidence before this study**

We searched PubMed for relevant articles published in English from database inception to Oct 15, 2021, using the terms “*Salmonella* Typhi”, “antimicrobial resistance”, “whole genome sequencing”, and “phylogeography analysis”. Several studies have explored the phenotypic and genotypic diversity of *Salmonella enterica* serovar Typhi (*S* Typhi) isolates using whole genome sequencing, and most of them involved small number of isolates from multiple countries. Two studies have described phylogeographical analysis of dominant lineages and identified transfers from Asia to Africa and an ongoing, multidrug-resistance epidemic within Africa.

**Added value of this study**

This study represents the largest genome sequencing study of *S* Typhi to date, with 3489 newly sequenced isolates from prospective surveillance studies in four of the highest typhoid burden countries in the world: Bangladesh, Nepal, Pakistan, and India. We combined these data with 4169 previously sequenced strains to characterise the emergence and geographical spread of antimicrobial resistant *S* Typhi. We applied bacterial phylodynamic methods to investigate how antimicrobial resistance influenced the population size of *S* Typhi, including displacement of less-resistant strains. Dated phylogenetic reconstruction and phylogeographic analyses were performed to estimate the frequency and location of antimicrobial resistance acquisition, along with dates of international spread. Additionally, our analysis also describes the emergence and evolutionary history of non-H58 lineages, about which relatively little is known.

**Implications of all the available evidence**

The results indicate that south Asia continues to be a crucial hub for *S* Typhi antimicrobial resistance acquisition, and antimicrobial-resistant clones that emerge in this region have been regularly introduced across borders within the region and intercontinentally. Our analysis also suggests that multidrug-resistant strains are declining in most parts of south Asia but are being replaced with strains containing ceftriaxone resistance (extensively drug-resistant), high-level fluoroquinolone resistance, or azithromycin resistance, which are reversing declines in the effective population size of *S* Typhi. These findings of frequent international spread and expansion of antimicrobial-resistant *S* Typhi strains underscore the importance of viewing typhoid control strategies through a global rather than country-specific lens.

Typhoid conjugate vaccines have proven effective for disease prevention, and WHO recommends introduction in countries with high burden of antimicrobial-resistant strains.^11^ However, given the current trajectory of antimicrobial resistance in *S* Typhi, waiting until a high burden of antimicrobial resistance is present within a country to introduce typhoid vaccines might be ill-advised. Understanding the historical emergence, and geographical spread of antimicrobial-resistant *S* Typhi might yield insights into where resistant strains might spread and how quickly they will become dominant.

Here, we leveraged prospective, population-based typhoid surveillance studies from four of the highest burden countries in south Asia: Bangladesh, India, Nepal, and Pakistan. We sequenced 3489 *S* Typhi organisms isolated over a 6-year period, and these data were combined with a global collection of more than 4000 additional genomes to investigate the emergence and geographical spread of antimicrobial-resistant *S* Typhi over the past 3 decades.

## Methods

### Bacterial isolates

This study included *S* Typhi isolates obtained from the Surveillance for Enteric Fever in Asia Project (SEAP; Bangladesh, Nepal, and Pakistan; 2016–19), Etiologies of Acute Febrile Illness Study (Nepal; 2014–16), and Surveillance for Enteric Fever in India Project (SEFI; 2017–19). The study methodologies have been previously described.^12^, ^13^ Participants included children and adults presenting to study sites with febrile illness. These sites included five health facilities in Dhaka, Bangladesh, 18 facilities across 16 cities in India, 11 facilities across three cities in Nepal, and two hospitals and a university laboratory network in Karachi, Pakistan. Among 9945 blood culture-confirmed typhoid cases, we selected a country-stratified sample of 3489 isolates for sequencing based on random (SEAP) or convenience sampling (SEFI). Sampling details are available in appendix 1 (p 2).

### Whole-genome sequencing and single-nucleotide polymorphism (SNP) analysis

Whole-genome sequencing was performed at the Wellcome Trust Sanger Institute (Hinxton, UK) using the Illumina HiSeq2500 platform (Illumina, San Diego, CA, USA), at a commercial Illumina service in Bangalore (India) and at the Wellcome Trust Research Laboratory in the Christian Medical College (Vellore, India) using the Illumina MiSeq platform (Illumina, San Diego, CA, USA). Paired-end reads were mapped to the *S* Typhi CT18 (AL513382) reference sequence using RedDog pipeline version V1beta.11. SNPs occurring in recombinant regions were detected by Gubbins version 2.4.1 and excluded.^14^ SNP data were used to assign genotypes using GenoTyphi version 1.9.1. To provide global context, additional *S* Typhi genomes^9^, ^15^, ^16^, ^17^, ^18^, ^19^, ^20^, ^21^, ^22^, ^23^, ^24^ were downloaded from The European Nucleotide Archive and subjected to the same SNP calling and recombination filtering pipeline.

### Phylogenetic analyses

Maximum likelihood phylogenetic trees were inferred from the SNP alignments using RAxML version 8.2.10.^25^ A generalised time-reversible model and a gamma distribution was used to model site-specific rate variation with 100 bootstrap pseudoreplicates used to assess branch support for the phylogeny. We selected the tree with the highest likelihood score as the best tree.

### Temporal and phylogeographical analysis

To investigate dates of emergence and geographical transfers, we inferred timed phylogenies. We used TempEst version 1.5 to assess temporal structure by conducting a regression of the root-to-tip branch distances of the tree as a function of the sampling time,^26^ which was confirmed by a clustered permutation test using R package BactDating version 1.1.0.^27^ For the non-H58 isolates, we estimated the best-fit models, tree topology, evolutionary rates, and phylogeography by using Bayesian Markov chain Monte Carlo method with BEAST2 version 2.6.2.^28^ Separate trees were fit for the most common non-H58 lineages (2.3.3, 2.5, 3.2.2, and 3.3). Isolates from each lineage were selected based on temporal, geographical, and phylogenetic diversity, as described in appendix 1 (p 3).

For the BEAST analysis, a general time reversible with gamma distribution model was selected, and sampling times (tip dates) were defined as the year of isolation. We tested support for a strict clock for each lineage using the relaxed clock test in the treedater R package version 0.5.0*,* and the strict clock was rejected in each instance.^29^ We therefore constructed time-phylogenies using coalescent exponential population priors with a relaxed clock (uncorrelated lognormal distribution).^5^, ^24^ Three independent runs were performed to ensure convergence. The effective sample sizes of the parameters were estimated to be more than 200 for all independent runs. Phylogeographical reconstruction was obtained by the continuous-time Markov chain process over discrete sampling locations.

For H58, we had 4761 isolates, which precluded temporal and phylogeographical analysis using BEAST due to computational constraints. To avoid significant down-sampling of isolates, we used the treedater R package^29^ with an uncorrelated, relaxed molecular clock to estimate the timed phylogeny using all available H58 sequences, which yielded a time of the most recent common ancestor matching a root-to-tip based analysis using BactDating.^5^ We reconstructed the ancestral state of nodes using the maximum parsimony approach with the Phangorn R package version 2.8.1, considering events with a location probability of more than 0·5 between connected nodes. For visualisation purposes, we selected a smaller subset of sequences to depict in a dated phylogenetic tree. For all phylogeographical analyses, we considered a geographical transfer when the most probable location between two connected nodes (or between a node and a tip) differed, and we considered the time window of transfer as the date range between the nodes (or between the node and tip). To investigate the geographical origin of H58 isolates, we evaluated the correlation between genetic diversity and geographical distances.^30^ To obtain stable estimates of the pairwise distance distribution, we included all countries with at least 20 sequences. We created a geographical grid of coordinates representing potential origins of H58 and fit weighted linear regression models relating log pairwise SNP distance with log geographical distance. We considered the most probable origin as that which had the highest coefficient of determination (R^2^). We fit separate models for Asia only and Asia and Africa, hypothesising that the relationship between diversity and distance might differ considering air travel across the Indian Ocean.

### Non-parametric phylodynamic inference of effective population size

To evaluate the historical effective population size for H58 lineage strains, we used the time-stamped H58 tree to estimate the effective population size through time using the skygrowth package version 0.3.1. We compared the effective population sizes of antimicrobial resistant and sensitive populations within countries. Epidemic success of antimicrobial resistant populations was also measured by comparing time-scaled haplotypic densities (THD; appendix 1 pp 3–4).

### Antimicrobial resistance associated gene detection and plasmid replicon analysis

Antimicrobial Resistance Identifier by Assembly (ARIBA) version 2.10.0 and the Comprehensive Antibiotic Resistance Database database version 1.1.8 were used to investigate antimicrobial resistance gene content. Point mutations in the QRDR of the DNA-gyrase *gyrA/B* and topoisomerase-IV *parC/E* genes, associated with reduced susceptibility to fluoroquinolones and quinolone resistance genes (*qnrS*), were also detected using ARIBA. Isolates were defined as multidrug-resistant if resistance genes were detected in the beta-lactam, trimethoprim–sulphonamide, and chloramphenicol classes. Plasmid replicons were identified using ARIBA and PlasmidFinder database.^30^

### Ethics statement

Ethical approvals were obtained from the Bangladesh Institute of Child Health Ethical Review Committee (01-02-2019), Christian Medical College Institutional Review Board (10393), Nepal Health Research Council (391/2018), Aga Khan University Hospital Ethics Committee and Pakistan National Ethics Committee (2019-0410-4188), Stanford University Institutional Review Board (39557), and US Centers for Disease Control and Prevention. Informed written consent was taken from adult participants and legal guardians of child participants.

### Role of the funding source

The funder of the study had no role in study design, data collection, data analysis, data interpretation, or writing of the report.

## Results

A total of 3489 *S* Typhi isolates collected between 2014 and 2019 were sequenced. Genotype analysis identified 29 distinct genotypes (appendix 1 p 5). Most isolates (2474 [70·9%]) belonged to genotype 4.3.1 (haplotype H58). We identified multiple, phylogenetically linked H58 sub-lineages shared across south Asia (appendix 1 p 6), most regularly between Bangladesh, Nepal, and India. Within the H58 isolates, 4.3.1.2 isolates formed distinct clades with intermingled isolates from India and Nepal, and 4.3.1.3 isolates, identified predominantly in Bangladesh, clustered with few isolates from India. By contrast, the H58 isolates from Pakistan largely clustered independently and was dominated by a monophyletic XDR clade (4.3.1.1.P1). Among non-H58 isolates, the most common subclades were 3.2.2 (190 [5·5%]), 3.3.2 (161 [4·6%]), 2.3.3 (140 [4·0%]), 2.5 (123 [3·5%]), and 3.3.1 (85 [2·4%]).

**Figure 1:**
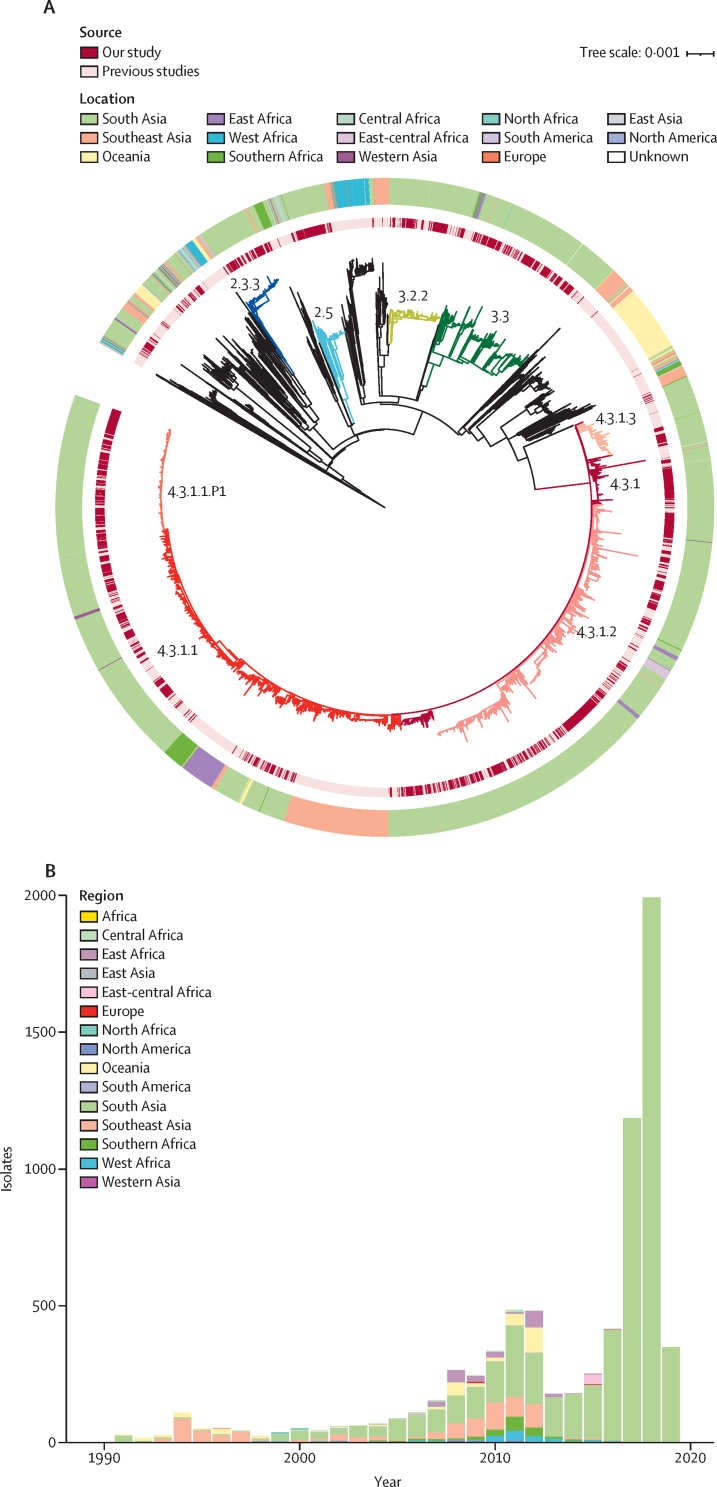
Global phylogeny of *Salmonella* Typhi (A) Maximum likelihood tree of 7658 *S* Typhi isolates from the global collection. Branch colours indicate the lineages 2.3.3 (blue), 2.5 (turquoise), 3.2.2 (yellow), 3.3 (green), 4.3.1 (dark red), 4.3.1.1 (red), 4.3.1.1.P1 (orange), 4.3.1.2 (pink), 4.3.1.3 (salmon), and other non-H58 (black). The inner ring indicates the source. The outer ring indicates the region of isolation. The scale bar indicates nucleotide substitutions per site. (B) Temporal distribution of sequenced *S* Typhi isolates by region.

To provide additional context for the 3489 new South Asian genomes, and better understand temporal and spatial distribution of lineages, we constructed a phylogeny incorporating an additional 4169 *S* Typhi sequences from organisms isolated from 1905 to 2018 from more than 70 countries (figure 1). Overall, the new sequences clustered with previously sequenced south Asian isolates, generating a distinct geographical structure. Genotype 4.3.1 formed a large subclade. Primary clades 2, 3, and 4 were distributed across continents with few isolates outside these clades. Notably, four subclades (2.3.3, 2.5, 3.2.2, and 3.3) were dominant in south Asia, accounting for 1239 (75·7%) of the 1634 non-H58 organisms.

We classified isolates as multidrug-resistant if they simultaneously contained genes conferring resistance to ampicillin (*bla*_TEM-1_), chloramphenicol (*catA1*), and trimethoprim–sulfamethoxazole (*dfrA7* plus *sul1* or *sul2*, or both). From 2000 onwards, we observed a declining trend in multidrug-resistant isolates in Bangladesh and India, a stable low proportion (30 [2·6%] of 1144) in Nepal, and an increasing proportion in Pakistan and Africa (appendix 1 p 7). Acquired resistance genes that contribute to the multidrug-resistant phenotype were identified in 2047 (26·8%) of the 7657 isolates of the global collection (appendix 1 p 21); of these 2016 (98·4%) were H58 isolates and 31 (1·6%) were non-H58 isolates. Among these non-H58 multidrug-resistant isolates, resistance was almost entirely plasmid-mediated (30 [96·8%] of 31). By contrast, for H58 isolates we observed that plasmid-mediated resistance was persistent in the H58 isolates in the 1990s, but from 2000 onward was less frequent, with most multidrug-resistant isolates containing chromosomal insertions of drug-resistance genes (1516 [75·2%] of 2016).

By contrast to the temporal trends in multidrug resistance, there was a consistent rise in the proportion of global *S* Typhi that were fluoroquinolone non-susceptible, primarily associated with mutations in *gryA, gyrB, parC,* and *parE* (appendix 1 p 7). The largest increase occurred in Bangladesh, exceeding 77 (98·7%) of 78 in 2008, followed by India in 2008 (32 [96·9%] of 33), Nepal in 2012 (35 [97·2%] of 36), and Pakistan in 2016 (84 [96·5%] of 87). Fluoroquinolone non-susceptible *S* Typhi increased from two (22·2%) of nine in 2006 to 52 (71·2%) of 73 by 2011 in southeast Asia. In Africa, this increase occurred more recently, starting around 2010. Overall, we found that QRDR mutations were significantly more common in the H58 isolates (4353 [77·5%] 5613) compared with other lineages (1257 [22·5%] of 5613; p<0·0001). From 2010 onwards, an increasing number of isolates had multiple QRDR mutations; more than 10% of all isolates had three mutations (appendix 1 p 8). Among the novel genomes, 437 were triple mutants (appendix 1 p 22), which are associated with high-level resistance to fluoroquinolones.^20^ Most (402 [92%] of 437) of these organisms occurred in H58 lineage II (4.3.1.2) in India and Nepal; the second most common (15 [3%] of 437) triple mutant genotype was 3.3, predominantly isolated in India. A comparison between genotypic and phenotypic resistance profile of all new isolates from south Asia are presented in appendix 1 (p 23).

Susceptibility to fluoroquinolones can be further reduced via plasmid-mediated acquisition of *qnr* genes. We identified *qnrS* in two non-H58 isolates and 686 H58 isolates that included genotype 4.3.1 (n=3), 4.3.1.1 (n=5), 4.3.1.P1 (n=550), and 4.3.1.3 (n=125). Most H58 isolates from Pakistan were XDR (4.3.1.P1) carrying the previously identified composite transposon containing *bla*_TEM-1_, *catA1, dfrA7, sul1, sul2* inserted in the chromosome, and *bla*_CTX-M-15_ and *qnrS* associated with an IncY plasmid.^9^ Azithromycin resistance, conferred by *acrB* mutations (Arg717Gln and Arg717Leu), was identified in 54 isolates across eight different genotypes including 4.3.1 (n=1), 4.3.1.1 (n=31), 4.3.1.2 (n=5), 4.3.1.3 (n=2), and non-H58 isolates comprising, genotype 2.3.3 (n=2), 3.2.2 (n=9), 3.3.2 (n=3), and 3.5.4 (n=1).

**Figure 2:**
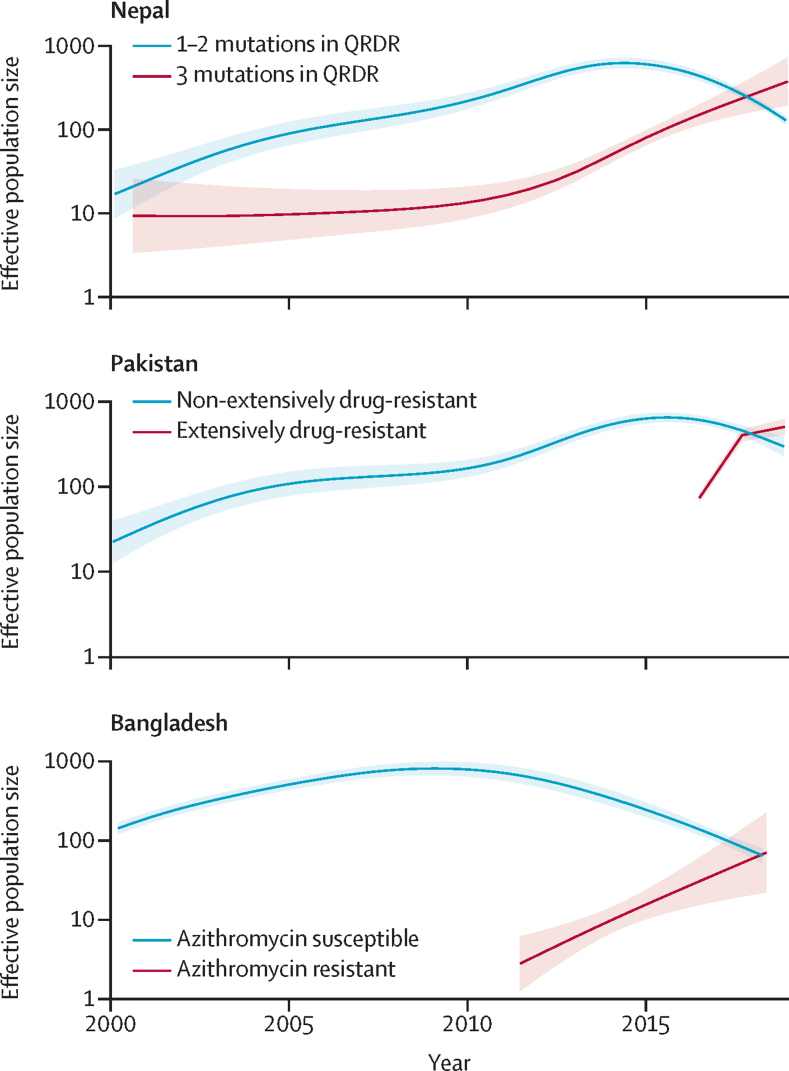
The effective population size of H58 lineages strains according to antimicrobial resistance genotype in Nepal, Pakistan, and Bangladesh. In Nepal, strains containing 1–2 mutations in the QRDR were compared with those containing three mutations. In Pakistan, XDR strains were compared with non-XDR strains. In Bangladesh, strains containing *acrB* mutations conferring azithromycin-resistance were compared with those not containing the mutations. Increased values indicate expanding effective population size. Light shading represents the 95% high probability density intervals of the estimates. QRDR=quinolone-resistance determining region. XDR=extensively drug-resistant.

To investigate how antimicrobial resistance has shaped the effective population size of *S* Typhi, we generated timed phylogenies and modelled the effective population size of antimicrobial susceptible and resistant organisms over time. To minimise the effect of location and lineage, we focused on the largest haplotype (ie, H58) and performed analyses within countries, evaluating key antimicrobial resistance determinants. In Nepal, we found that the effective population size (N_e_) of *S* Typhi containing one or two QRDR mutations rose steadily from 2000, beginning to decline from 2017, and triple mutants have steadily increased from 2010 (figure 2). In Pakistan, the N_e_ of non-XDR H58 *S* Typhi increased from 2000 until around 2015 and began to fall; XDR organisms emerged and have been rapidly growing in frequency since 2016, eclipsing the effective population of non-XDR organisms by 2018. In Bangladesh, the N_e_ of H58 had slowly declined from around 2010; however, azithromycin-resistant organisms emerged in 2013 with a corresponding increase in N_e_. In all three settings, organisms with key antimicrobial resistance conferring mutations or genes appear to be replacing their susceptible (or, in the case of fluoroquinolones, less-resistant) counterparts. Additionally, we measured the epidemic success of antimicrobial resistant populations by comparing THD. We found that the THD success index was higher in QRDR triple mutant isolates than those containing 1–2 mutations (p<0·0001) or none (p<0·0001; appendix 1 p 9). We also found a significant positive association between THD and XDR strains (p<0·0001; appendix 1 p 10).

**Figure 3:**
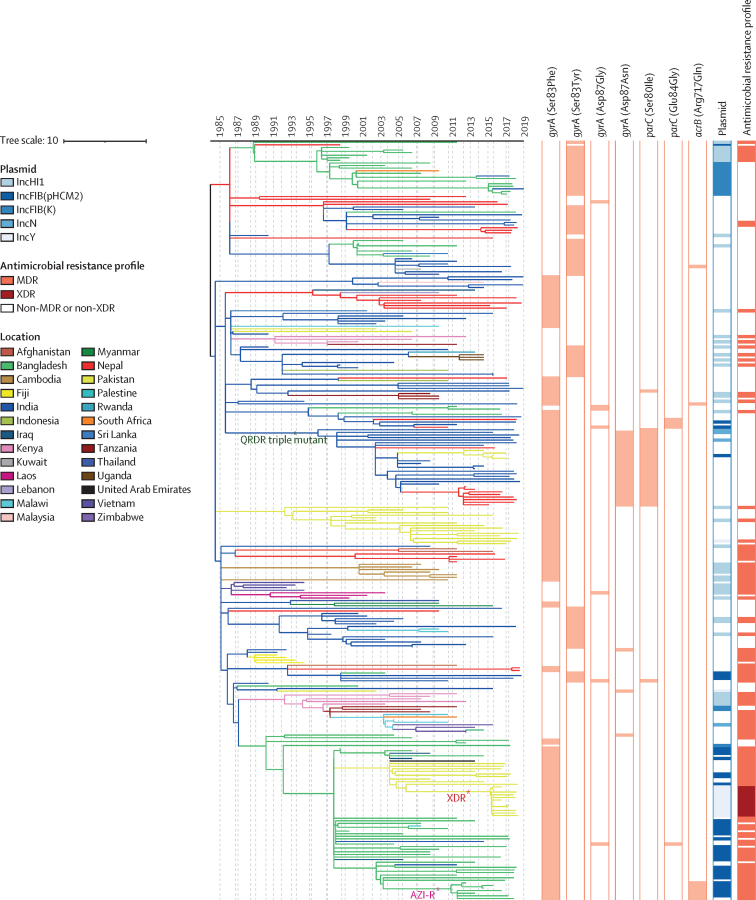
Phylogeography and global expansion of genotype 4.3.1 (H58) *Salmonella* Typhi isolates Timed phylogenetic tree of genotype 4.3.1 *S* Typhi isolates. The branch lengths are scaled in years and are coloured according to the location of the most probable ancestor of descendant nodes. The scale bar indicates nucleotide substitutions per site. AZI-R=azithromycin resistant. MDR=multidrug resistant. QRDR=quinolone-resistance determining region. XDR=extensively drug-resistant.

**Figure 4:**
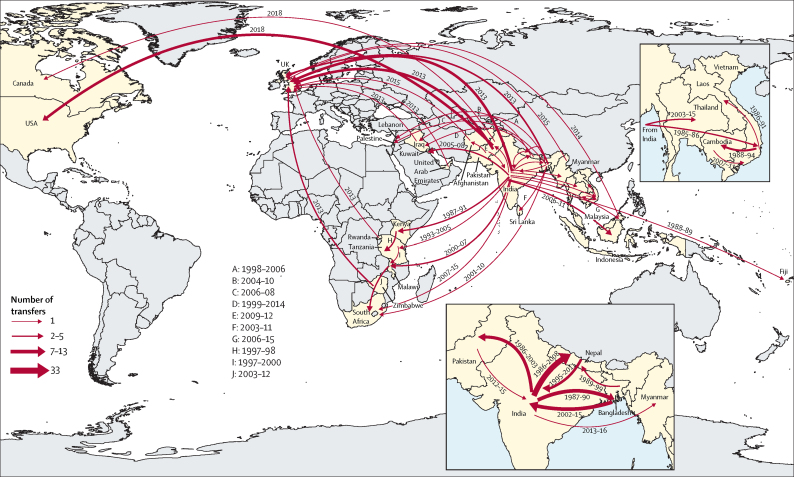
Geographical transfers within lineage 4.3.1 (H58) inferred from ancestral state reconstruction of the timed phylogenetic tree The size of each arrow is scaled to the estimated number of transfers between the countries. Dates indicate the estimated first transfer between each pair of countries.

Using country of sampling as a discrete trait, we generated dated phylogenies to reconstruct the evolutionary history and geographical spread of H58 lineage and the four common non-H58 genotypes. Phylogeographical reconstruction of H58 isolates estimated that the time of most recent common ancestor of all contemporary H58 strains existed around 37 years ago (1984). The distribution of isolates and tree topology are consistent with at least 138 international transfer events, including multiple introductions within south Asia and dissemination from south Asia into southeast Asia and Africa, as well as many travel-related cases identified in the UK and USA (figure 3; figure 4). We also predicted that ciprofloxacin-resistant triple mutant isolates most probably originated in India around 1996 and were introduced into Pakistan between 2005 and 2013 and into Nepal on at least three occasions (2003–15). We identified frequent transmissions of international transfer of multidrug-resistant isolates (n=33), with multiple introductions from south Asia to southeast Asia and Africa followed by local expansion.

Our models assessing pairwise diversity in H58 isolates and geographical distance identified India as the most probable origin, in both the model including Asian and African isolates and the model restricted to Asian isolates. We found moderate support for a log-linear relationship between pairwise diversity and distance in the model including Asian and African isolates (R^2^=0·72) but strong correlation (R^2^=0·90) in the model of diffusion within Asia (appendix 1 pp 11–12).

The major non-H58 clades also acquired antimicrobial resistance loci and spread within and from south Asia. Genotype 2.3.3 circulated predominantly in Bangladesh but spread to Pakistan and India within the past 30 years (appendix 1 pp 13–14). Genotype 2.5, which might have circulated in India for more than 100 years (appendix 1 pp 15–16), has been transferred to sub-Saharan Africa and Nepal multiple times, including two instances with strains containing QRDR mutations since 2015. Genotype 3.2.2 organisms originating from Bangladesh were observed in south Asia only. We observed a single instance of transfer from Bangladesh to Nepal and ongoing local expansion. By contrast, we found that these organisms have been regularly transferred from Bangladesh to India (appendix 1 pp 17–18). Transfer events included at least four recent introductions of fluoroquinolone non-susceptible organisms between 2006 and 2017. The most recent common ancestor of genotype 3.3 was estimated to have been from India more than 200 years ago (appendix 1 pp 19–20), but moved extensively across south Asia, establishing large subclades in Bangladesh and Nepal, before progressing to sub-Saharan Africa, the Middle East, and southeast Asia. Genotype 3.3 organisms with QRDR mutations have moved from India to Nepal on multiple occasions.

**Figure 5:**
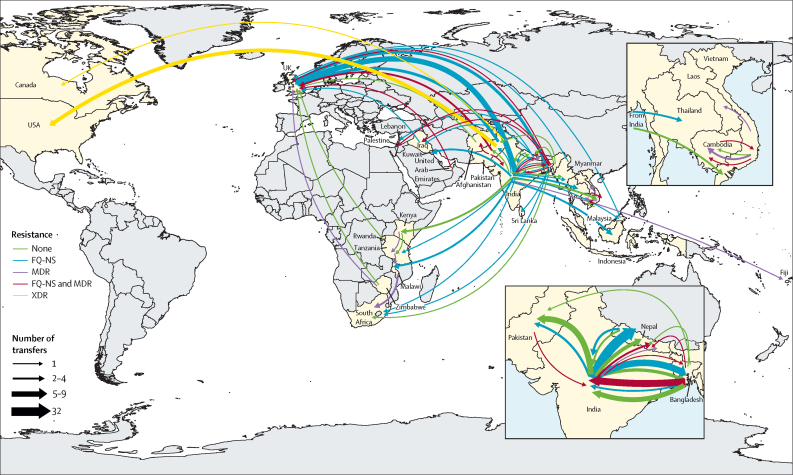
Major geographical transfers from 1990 onwards within the non-H58 and H58 lineages, inferred from the phylogenetic trees The size of each arrow indicates the relative number of probable transfers between the countries. Arrow colours indicate antimicrobial resistance pattern. FQ-NS=Fluoroquinolone non-susceptible. MDR=multidrug resistant. XDR=extensively drug resistant.

Overall, our analysis identified evidence for at least 197 introduction events between countries, of which 138 were intracontinental and 59 were intercontinental (figure 5). The most common international transmission events were within south Asia and from south Asia to southeast Asia, east Africa, and southern Africa. We estimated that resistance-conferring mutations to fluoroquinolones (n=94) or azithromycin (n=7) have independently emerged on at least 101 separate occasions within the past 30 years, mostly in south Asia (n=94), and occasionally arising in southeast Asia, Africa, and South America. Additionally, isolates carrying QRDR mutations were transferred between countries on at least 119 independent occasions.

## Discussion

This analysis of *S* Typhi genome sequences reveals that acquisition of antimicrobial resistance through plasmids or homoplastic mutations has occurred frequently across multiple lineages and been accompanied by expansion and international spread of antimicrobial-resistant *S* Typhi clones. We identified numerous international and intercontinental transfers of *S* Typhi over the past 30 years, with the majority associated with antimicrobial resistance. Once introduced to a new setting, antimicrobial-resistant *S* Typhi became quickly fixed, as broadly exemplified with fluoroquinolone non-susceptible clades in multiple countries and XDR *S* Typhi in Pakistan. This rapid emergence, spread, and fixation of antimicrobial resistance suggests that making decisions regarding typhoid conjugate vaccine introduction based on current antimicrobial resistance data might miss a crucial window for prevention. Specifically, we found that south Asia continues to be an important hub for the generation of antimicrobial resistance and that the clones emerging here regularly move internationally, underscoring the need for resources to support typhoid control in this region.

Our ancestral state reconstruction and analysis of diversity loss over distance both identified the Indian subcontinent as an origin for most *S* Typhi lineages. Our analysis of diversity and distance from the hypothetical geographical origin (in India) found support for a log-linear relationship between diversity and distance within Asia, although model fit declined when incorporating samples from Africa, probably due to the role of air travel in the spread of *S* Typhi from south Asia to east Africa. The spread of *S* Typhi within and from south Asia might be linked to migration patterns, both between countries in south Asia and to other regions such as east Africa and southeast Asia, where *S* Typhi was able to spread due to poor water and sanitation infrastructure. The relationship between the Indian diaspora and spread of H58 strains has been previously recognised.^31^

A 2015 study investigated the emergence and global spread of the dominant H58 lineage in *S* Typhi, and a subsequent analysis examined the spread of H58 and 3.1.1 in sub-Saharan Africa.^5^, ^24^ Our findings affirm and extend these findings with a much larger set of sequences, characterising the evolutionary history and phylogeography of H58 and four major non-H58 sublineages. Leveraging advances in maximum likelihood-based timed phylogenetic reconstruction enabled us to incorporate far more sequences in the temporal and phylogeographical analysis (eg, 4761 strains compared with 114 strains in the study by Wong and colleagues^5^). This analysis in turn provided a higher resolution window into timing and location of antimicrobial resistance emergence, as well as the geographical spread of *S* Typhi. Furthermore, the application of phylodynamic methods enabled quantification of the effects of antimicrobial resistance emergence on the population size of the dominant *S* Typhi lineage, providing new evidence that antimicrobial resistance facilitates its spread and, in some contexts, reversed trends of declining *S* Typhi populations.

Our data are consistent with studies suggesting that multidrug-resistant *S* Typhi (strains resistant to the classical first line drugs) is now generally on the decline in south Asia.^32^, ^33^, ^34^ The decline of multidrug-resistant *S* Typhi in Asia has been accompanied by a decrease in the proportion of isolates carrying IncHI1 plasmids (except for Pakistan, where the multidrug-resistant decline reversed amid the emergence of the XDR lineage). In our study, multidrug resistance was principally associated with H58 carrying chromosomally integrated antimicrobial resistance genes. The integration of antimicrobial resistance genes into the *S* Typhi chromosome remains a concern, as it provides a mechanism for stable vertical transmission of multidrug-resistant phenotypes.^5^, ^35^ By contrast to south Asia, multidrug-resistant typhoid associated with H58 and non-H58 isolates appears to be increasing in parts of Africa, with outbreaks being reported in the past decade.^24^, ^36^ QRDR mutations have independently arisen frequently in all *S* Typhi lineages. Nearly all sustained clones containing QRDR mutations appear to have arisen in south Asia, and many have spread regionally and globally. Notably, our analysis revealed that highly fluoroquinolone-resistant *S* Typhi triple mutants have recently emerged in six different genotypes. Our phylogeographical analysis suggests that these clones most probably originated in India and disseminated to neighbouring countries including Nepal and Pakistan.

The emergence and spread of resistance to third-generation cephalosporins and azithromycin in the past decade further complicates typhoid fever treatment.^8^, ^9^ By 2019, within 3 years of its first recognition, the XDR genotype (4.3.1.1.P1) became the dominant genotype in Pakistan. At present, all XDR *S* Typhi strains identified have been susceptible to azithromycin and carbapenems.^37^ Concerningly, azithromycin-resistant *S* Typhi have recently been reported in Bangladesh, India, Pakistan, Nepal, and Singapore,^10^, ^38^, ^39^ arising from mutations in *acrB*. These mutations have arisen independently multiple times in distinct lineages.^10^ To date, XDR *S* Typhi isolates containing mutations in *acrB* have not yet been identified. Such organisms would preclude effective treatment with established oral antimicrobials, which could lead to increased hospitalisation rates and potentially greater morbidity and mortality.

Our findings should be interpreted within the context of the limitations of the available data. Although this analysis included the largest collection of novel *S* Typhi genomes to date, there remains underrepresentation of sequences from several regions, including disproportionately small numbers from many countries in sub-Saharan Africa and Oceania where typhoid is endemic. This underrepresentation constrains the inference of timed phylogenies and ancestral state reconstruction; more sequences from these regions are needed to improve our understanding of timing and patterns of spread. Even in countries with more dense sampling, most isolates were derived from a small number of surveillance sites and might not be representative of the distribution of circulating strains. Because *S* Typhi genomes only cover a fraction of all typhoid fever cases, phylogenies are highly incomplete; our estimates for antimicrobial resistance-conferring homoplastic mutations and international transfers represent lower bounds might substantially underestimate their true frequency. These circumstances highlight the need for expanding genomic surveillance to provide a more comprehensive window into the emergence, expansion, and spread of antimicrobial-resistant organisms.

This study highlights the emergence and geographical spread of antimicrobial-resistant *S* Typhi, with evidence of frequent international exportation. This observation underscores the importance of approaching typhoid fever control and antimicrobial resistance as a global rather than local problem. The recent emergence of XDR and azithromycin-resistant *S* Typhi creates greater urgency for rapidly expanding prevention measures, including use of typhoid conjugate vaccines in typhoid-endemic countries. Such measures are needed in countries where antimicrobial resistance prevalence among *S* Typhi isolates is currently high, but given the propensity for international spread, should not be restricted to such settings.

## Data sharing

Illumina sequence data was submitted to the European Nucleotide Archive. Sequence data from 4169 *S* Typhi strains from previous studies were also included for global context raw sequence data for these isolates are available in European Nucleotide Archive. Details and individual accession numbers of sequence data included in our analysis have been included in appendix 2 and appendix 3.

## Declaration of interests

IB has consulted for BlueDot, a social benefit corporation that tracks the spread of emerging infectious diseases. SPL reports travel fees from the Bill & Melinda Gates Foundation. ZAD is a coordinator and founding member of the Global Typhoid Genomics Consortium and reports grants from EU Horizon 2020 for typhoid research outside the submitted work. All other authors declare no competing interests.
