# Supplementary material for: The international and intercontinental spread and expansion of antimicrobial-resistant Salmonella Typhi: a genomic epidemiology study
Source: Lancet Microbe. 2022 Aug;3(8):e567–77. doi: 10.1016/S2666-5247(22)00093-3 (PMC9329132; doi:10.1016/S2666-5247(22)00093-3)
Supplement: Supplementary appendix 1 [file mmc1.pdf]

THE LANCET
Microbe

Supplementary appendix 1

This appendix formed part of the original submission and has been peer reviewed. We post it as supplied by the authors.

Supplement to: da Silva K E, Tanmoy A M, Pragasam A K, et al. The international and intercontinental spread and expansion of antimicrobial-resistant *Salmonella* Typhi: a genomic epidemiology study. *Lancet Microbe* 2022; published online June 21. https://doi.org/10.1016/S2666-5247(22)00093-3.

International and intercontinental spread and expansion of antimicrobial-resistant *Salmonella* Typhi: a genomic epidemiology study

Supplementary appendix

Table of Contents

Supplementary Methods **.......... 2**

Antimicrobial susceptibility profiles 2

Sampling of SEAP and SEFI isolates for sequencing **.......... 2**

Whole genome sequencing and Bioinformatics **.......... 2**

Filtering the SNPs **.......... 3**

Non-H58 strain selection for BEAST analyses **.......... 3**

Time-scaled haplotypic density 3

Supplementary references 4

Supplementary Figure 1 5

Supplementary Figure 2 6

Supplementary Figure 3 7

Supplementary Figure 4 8

Supplementary Figure 5 9

Supplementary Figure 6 13

Supplementary Figure 7 13

Supplementary Figure 8 13

Supplementary Figure 9 15

Supplementary Figure 10 17

Supplementary Figure 11 19

Supplementary Table 3 22

Supplementary Table 4 22

Supplementary Table 5 22

Supplementary Table 6 24

Supplementary Methods

Antimicrobial susceptibility profiles

Specimens from positive blood culture bottles were sub-cultured on MacConkey agar plate and incubated at 37˚C for 18–24 hours. *S*. Typhi colonies were identified using standard biochemical test. Antimicrobial susceptibility for ampicillin, co-trimoxazole, chloramphenicol, ciprofloxacin and ceftriaxone were determined using the disk diffusion method (Oxoid, Thermo Scientific, MA, USA). All zone diameter was interpreted according to EUCAST v8.0 clinical breakpoints.

Sampling of SEAP and SEFI isolates for sequencing

Bangladesh

A total of 820 *S*. Typhi isolates were selected for sequencing; 80 were selected for suspected resistance to azithromycin, and 740 isolates were selected from a library of 2,366 *S*. Typhi isolates using a stratified random selection procedure to sample in proportion to the distribution of samples by study year, age and sex. All isolates were collected from October, 2016 to July, 2018.

India

In India, isolates were obtained from 12 sites in the Surveillance for Enteric Fever in India project, which included prospective cohort studies and hospital-based surveillance. Sites were selected to be geographically representative, covering four zones (East, West, North and South). We selected 993 *S*. Typhi consecutive isolates from SEFI, obtained between October, 2017 and December, 2019.

Nepal

In Nepal, isolates were obtained from two studies: the Fever Etiologies Study (2014-2017) and SEAP (2017-2019). Surveillance was performed at hospitals and clinics in urban (Kathmandu), peri-urban (Kavrepalanchok) and rural areas, but the majority of typhoid cases (and isolates) were obtained from Kathmandu and Kavrepalanchok. We selected 816 randomly selected isolates spanning this study period for sequencing.

Pakistan

In Pakistan, isolates were obtained as part of SEAP from Aga Khan University Hospital and its affiliated laboratory network and from Kharadar General Hospital, both in Karachi. We selected a consecutive 860 isolates for sequencing, obtained between October, 2016 and December, 2018.

Whole genome sequencing and Bioinformatics

Sequence data quality was checked using FastQC v0.11.9 to remove low quality reads (1). We summarized all quality indicators using MultiQC v1.7 (2). Species identification was confirmed with Kraken2 (3), and the

Salmonella in silico Typing Resource (SISTR) was used for WGS-based serotyping (4). Short Read Sequence Typing for Bacterial Pathogens (SRST2) (5) was used to map known alleles and identify MLSTs directly from reads according to the *Salmonella enterica* MLST scheme (https://pubmlst.org/salmonella/).

Filtering the SNPs

RedDog mapping pipeline v1beta.11 (https://github.com/ katholt/reddog) was used for mapping and SNP calling. RedDog uses Bowtie2 v2.4.1 (6) was used to map reads to the reference genome and SAMtools v1.10 to identify SNPs that have a phred quality score above 30, and to filter out those SNPs supported by less than five reads, or with 2.5x the average read depth that represent putative repeated sequences, or those that have ambiguous base calls. For each SNP that passes these criteria in any one isolate, consensus base calls for the SNP locus were extracted from all genomes mapped, with those having phred quality scores under 20 being treated as unknown alleles and represented with a gap character.

Chromosomal SNPs with confident homozygous calls (phred score above 20) in >95% of the genomes mapped (representing a 'soft' core genome) were concatenated to form an alignment of alleles using the RedDog python script parseSNPtable.py with parameters -m cons, aln and -c 0.95 and SNPs called in prophage regions and repetitive sequences (354 kb; ~7.4% of bases) in the CT18 reference chromosome, as defined previously (7) were excluded to form an alignment of 14,901 variant sites. SNPs occurring in recombinant regions were excluded resulting in a final alignment of 11,978 chromosomal SNPs. Genome ssequences from the global vollection were subjected to both SNP calling, and recombination filtering as described above, resulting in an alignment of 28,897chromosomal SNPs.

Non-H58 strain selection for BEAST analyses

Strains were selected to cover the full temporal (1905-2019) and geographical range of non-H58 lineages. For 2.3.3, 2.5, and 3.2.2 lineages, we selected all the isolates reported before our study. Isolates described in our study were selected according to the clades and branches of the maximum likelihood tree, to ensure we were including isolates with as much diversity as possible. For 3.3 lineage, we selected at least one isolate for each country and year of each sub-lineage. For countries with a large number of samples, we selected a maximum of three isolates for each country and year. From SEAP/SEFI isolates, we selected isolates according to the clades and branches of the maximum likelihood tree, with a maximum of 10 per year of each 3.3 sub-lineage.

Time-scaled haplotypic density

We measured the epidemic success of antimicrobial resistant populations using the time-scaled haplotypic density (THD) method. THD computes an independent index of success for each isolate in a collection and allows us to identify independent predictors of success (8). To minimize the effect of location and lineage, we focused on H58 isolates and performed analyses within countries. In this context, we illustrate the use of THD to address the question of whether the presence of multiple QRDR mutations has a measurable impact on pathogen success at

population scale. THD estimates were computed from the matrix of pairwise SNP distances using a timescale of 10 years, an effective genome size of 4.5×10^6 bp and an average per-nucleotide substitution rate of 7.98×10^{-6} as estimated in our analysis. We performed the same analysis to compare non-XDR and XDR H58 *S.* Typhi organisms in Pakistan. THD estimates were using a timescale of tMRCA50 years. For the analysis we used a range of tMRCA50s (5, 10, 20 for Nepal; 3, 5, 10 for Pakistan) and obtained the same results.

Supplementary references

1. Andrews S. Babraham Bioinformatics - FastQC A Quality Control tool for High Throughput Sequence Data. Soil. 1973.
2. Ewels P, Magnusson M, Lundin S, Käller M. MultiQC: Summarize analysis results for multiple tools and samples in a single report. Bioinformatics. 2016;
3. Wood DE, Lu J, Langmead B. Improved metagenomic analysis with Kraken 2. Genome Biol. 2019;
4. Yoshida CE, Kruczkiewicz P, Laing CR, Lingohr EJ, Gannon VPJ, Nash JHE, et al. The salmonella in silico typing resource (SISTR): An open web-accessible tool for rapidly typing and subtyping draft salmonella genome assemblies. PLoS One. 2016;
5. Inouye M, Dashnow H, Raven LA, Schultz MB, Pope BJ, Tomita T, et al. SRST2: Rapid genomic surveillance for public health and hospital microbiology labs. Genome Med. 2014;
6. Langmead B, Salzberg S. Bowtie2. Nat Methods. 2013;
7. Wong VK, Baker S, Connor TR, Pickard D, Page AJ, Dave J, et al. An extended genotyping framework for Salmonella enterica serovar Typhi, the cause of human typhoid. Nat Commun. 2016;
8. Wirth T, Wong V, Vandenesch F, Rasigade J-P. Applied phyloepidemiology: Detecting drivers of pathogen transmission from genomic signatures using density measures. Evol Appl [Internet]. 2020 Jul 1;13(6):1513–25. Available from: https://doi.org/10.1111/eva.12991

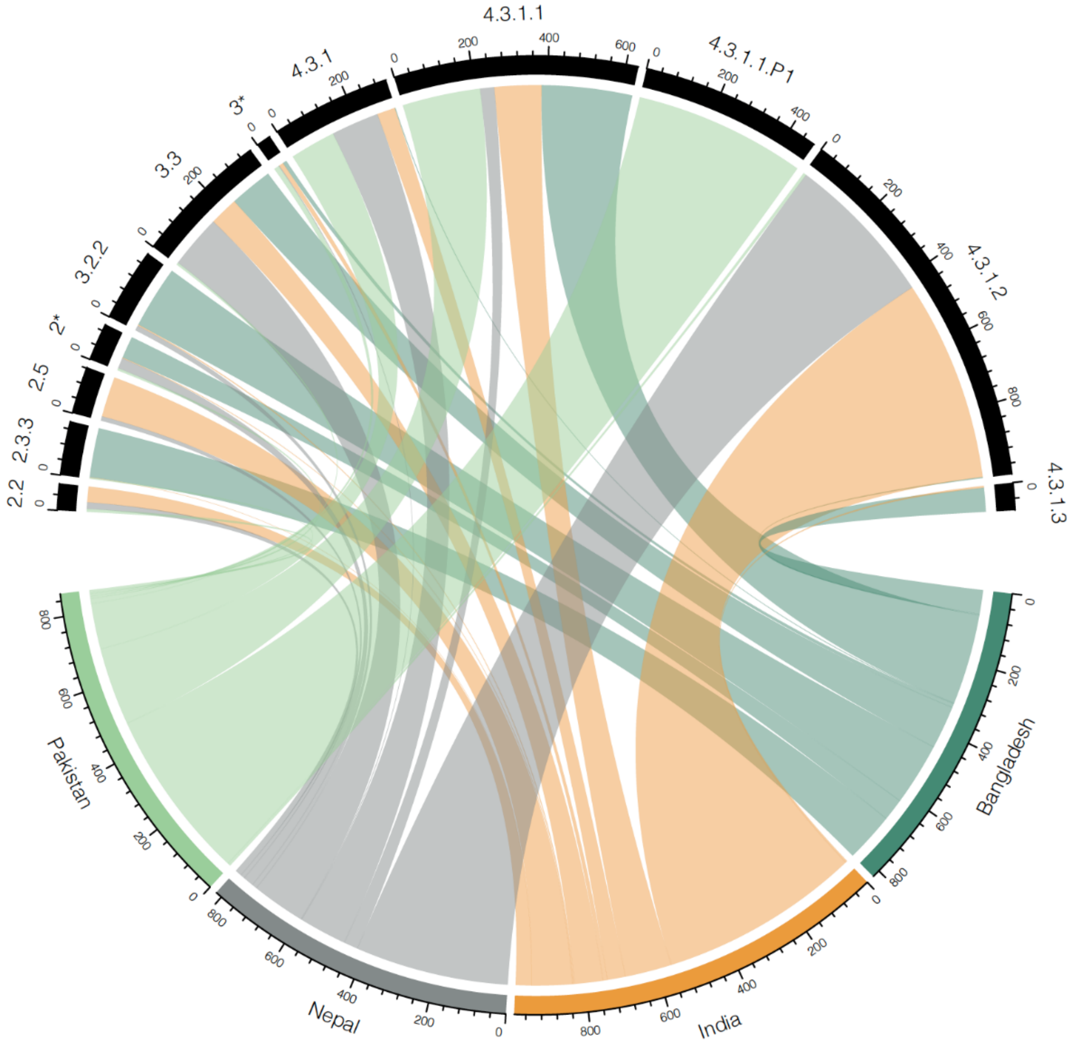

Supplementary Figure 1. Frequency and distribution of genotypes of contemporary *Salmonella* Typhi isolates included in our study (2014 to 2019). All the genotypes present in in less than three samples are not shown. 2* includes 2, 2.0.1, 2.0.2, 2.1.7, 2.2.1, 2.2.2, 2.2.4, 2.3.4 and 2.4 genotypes. 3* includes 3, 3.0.1, 3.1.2 and 3.2.1 genotypes.

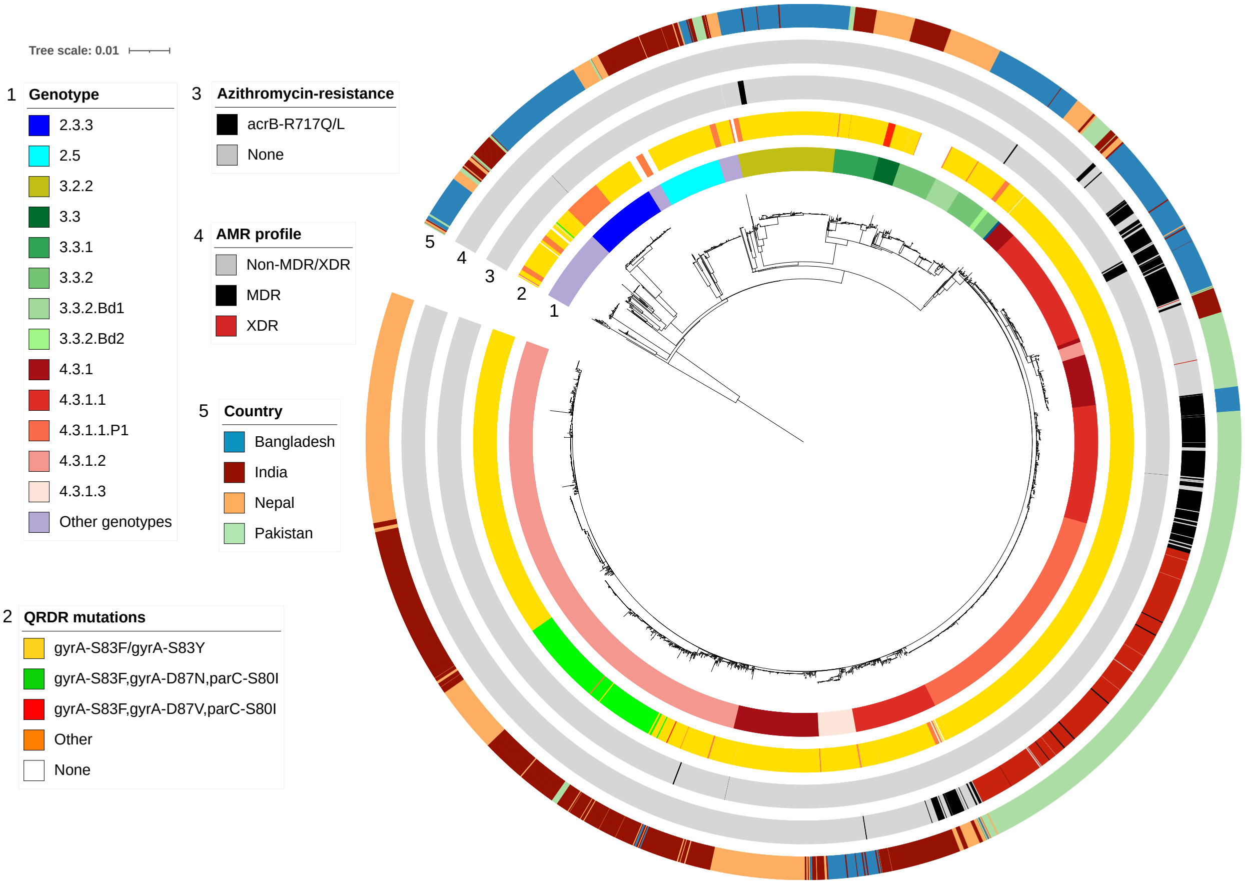

Supplementary Figure 2. Maximum likelihood tree of 3,489 contemporary *S*. Typhi isolates from South Asia. Inner ring indicates the genotypes. The second ring indicates QRDR mutations profiles. Third ring indicates the presence of *acrB* mutations associated with azithromycin-resistant isolates. Fourth ring indicates MDR or XDR status, and the outer ring indicates country of isolation. The scale bar indicates nucleotide substitutions per site.

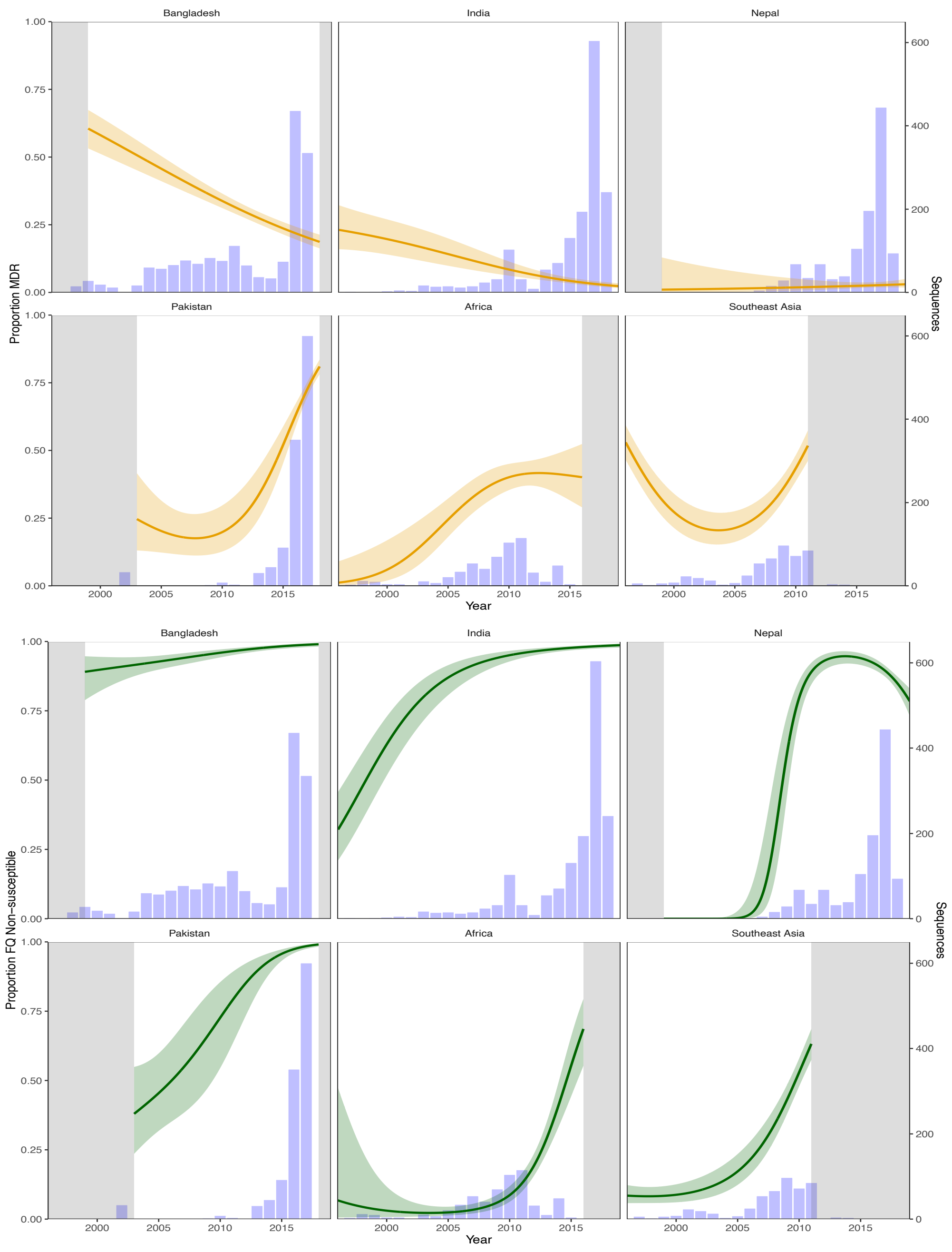

Supplementary Figure 3. Antimicrobial resistance trends of *Salmonella* Typhi isolates. **(a)** Proportion and temporal distribution of multidrug-resistant (MDR) *S*. Typhi isolates from our global collection. Isolates were considered MDR if they contained genes conferring resistance to chloramphenicol (*catA1*), trimethoprim-sulfamethoxazole (*dfrA7*, *sul1*, or *sul2*) and ampicillin (*bla*$_{\text{TEM-1}}$). Shading indicates missing data. Bars indicate temporal distribution of isolates. **(b)** Proportion and temporal distribution of Fluoroquinolone non-susceptible (FQ-NS) *S*. Typhi isolates from our global collection. We classified isolates as FQ-NS if they carried quinolone resistance genes (*qnrS*), and/or point mutations in the quinolone resistance determining region (QRDR) of the DNA-gyrase *gyrA/B* and topoisomerase-IV *parC/E***.**

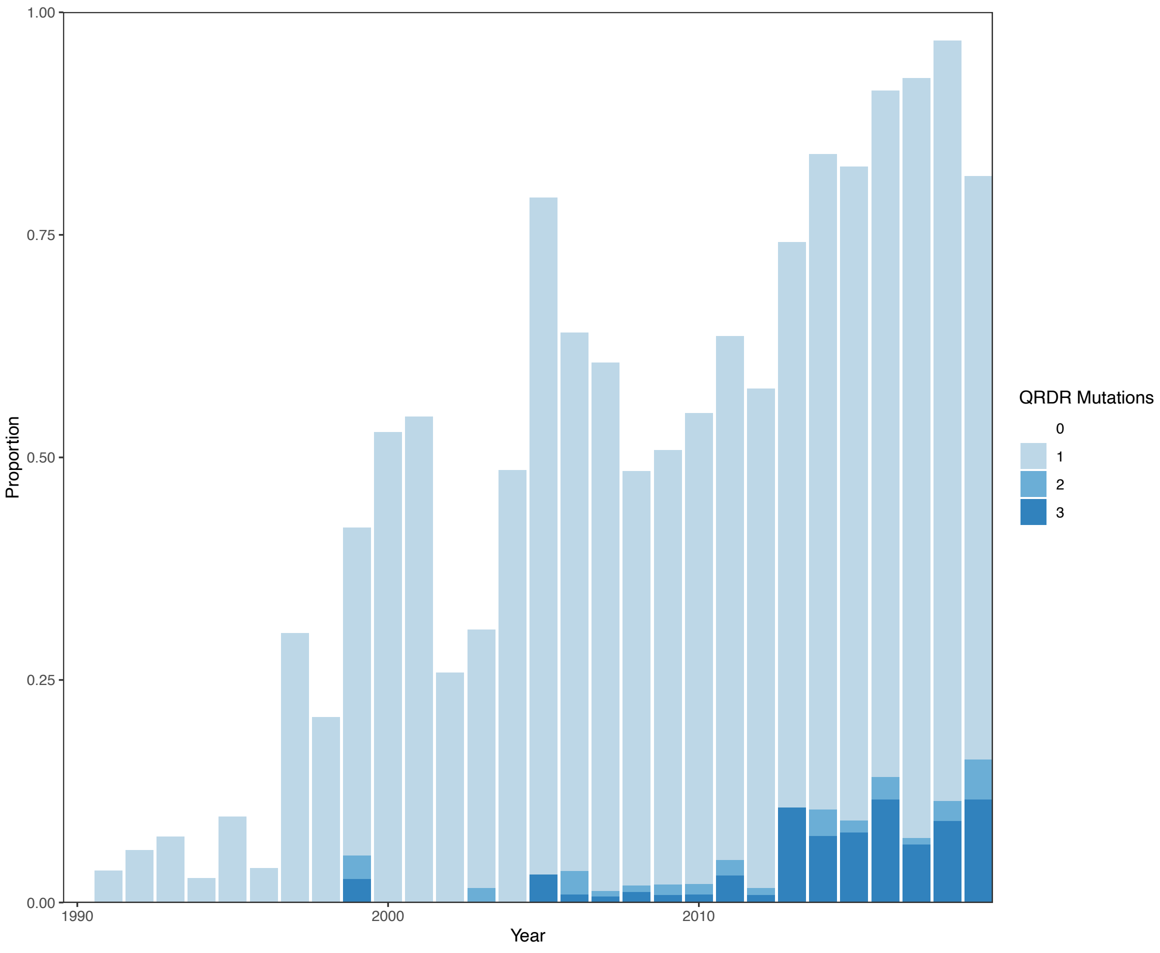

Supplementary Figure 4. Frequency of chromosomal point mutations in the quinolone resistance-determining region (QRDR) per isolate over time.

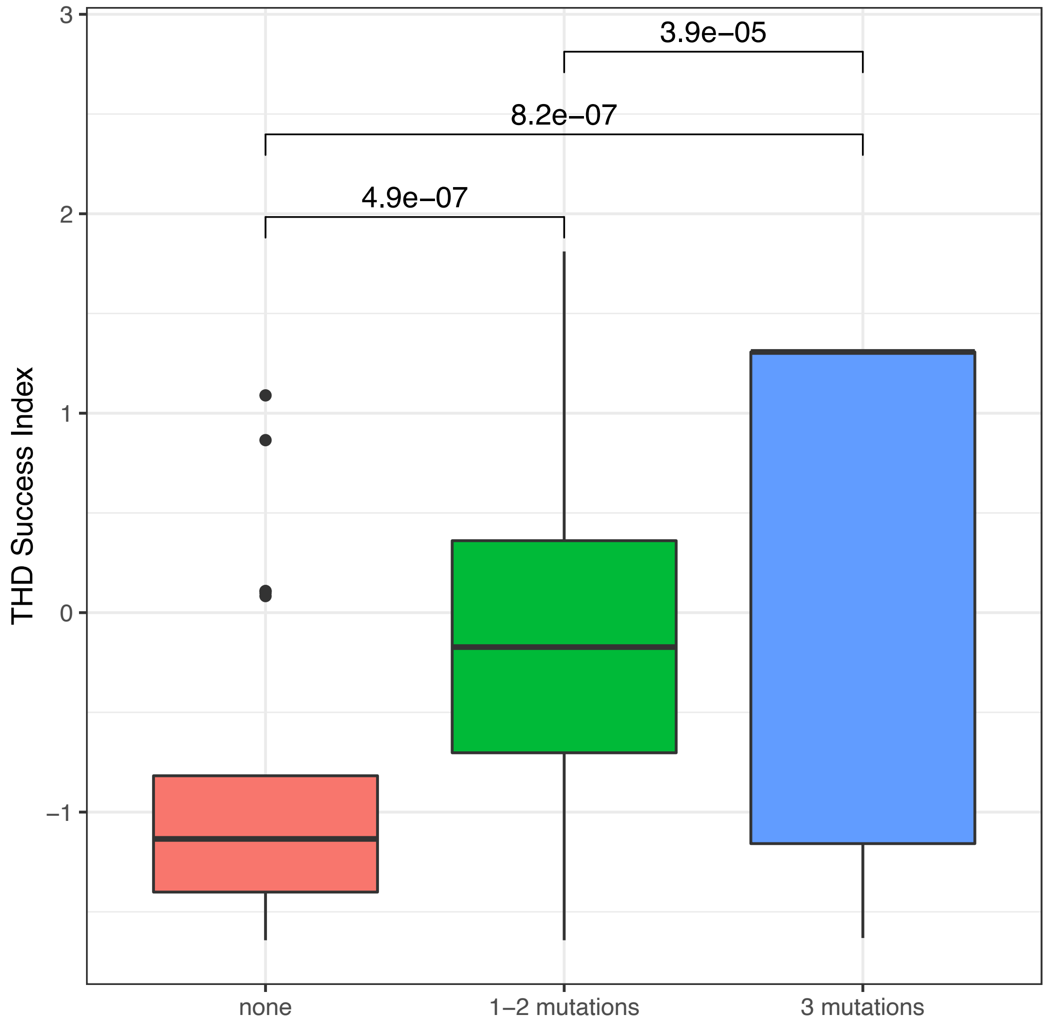

Supplementary Figure 5. Distribution of the THD success index in H58 *S.* Typhi isolates according to AMR profile. (**a**) THD in isolates from Nepal, stratified by number of mutations in the Quinolone resistance determining region (QRDR).

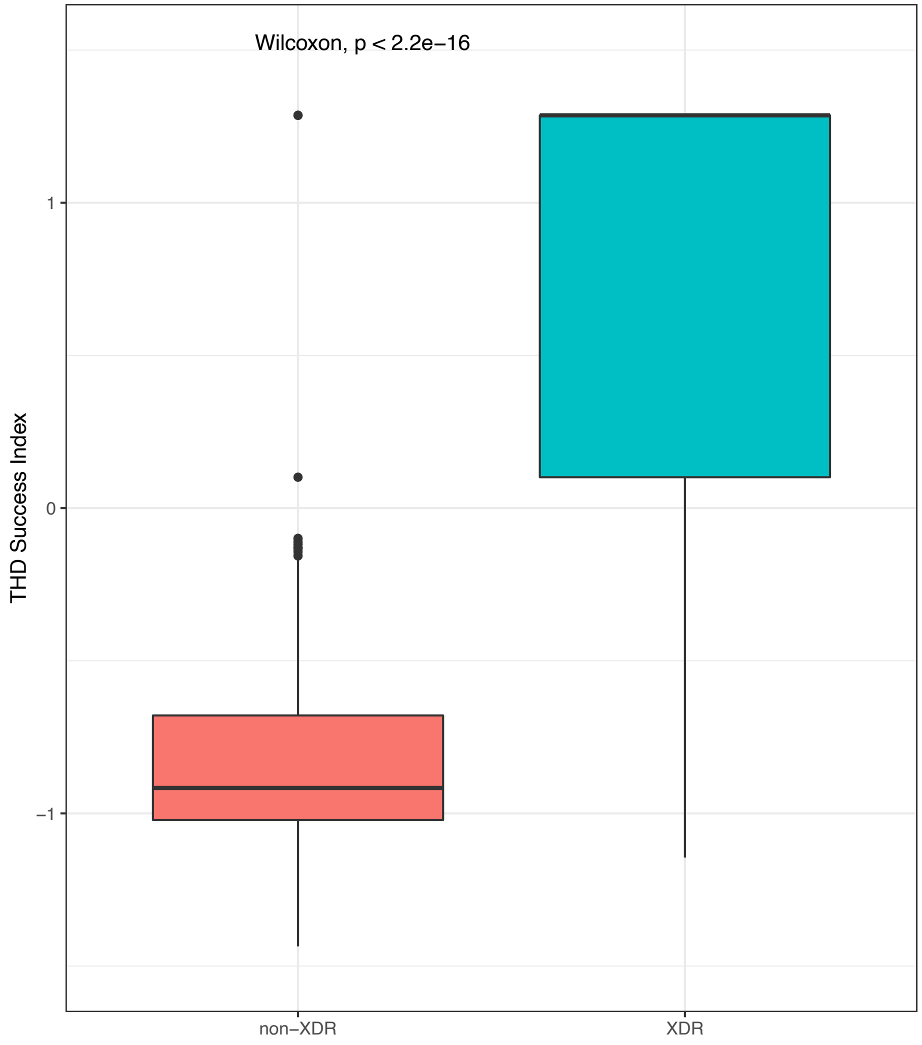

(b) THD in isolates from Pakistan, stratified by whether the strain was extensively drug resistant (XDR) or not.

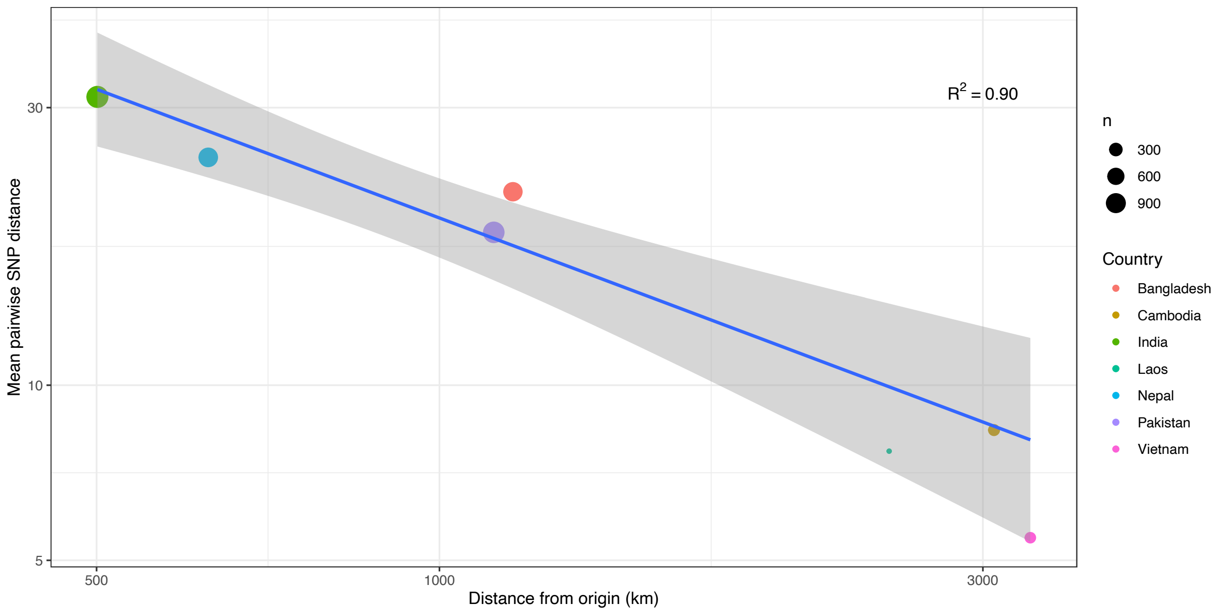

Supplementary Figure 6. Genetic diversity and distance from the geographic origin of H58 *S.* Typhi restricted to Asian isolates. (a) Correlation between geographic distance (log km^2) and genetic diversity (log mean pairwise distance) for the best supported origin. n represents the number of samples.

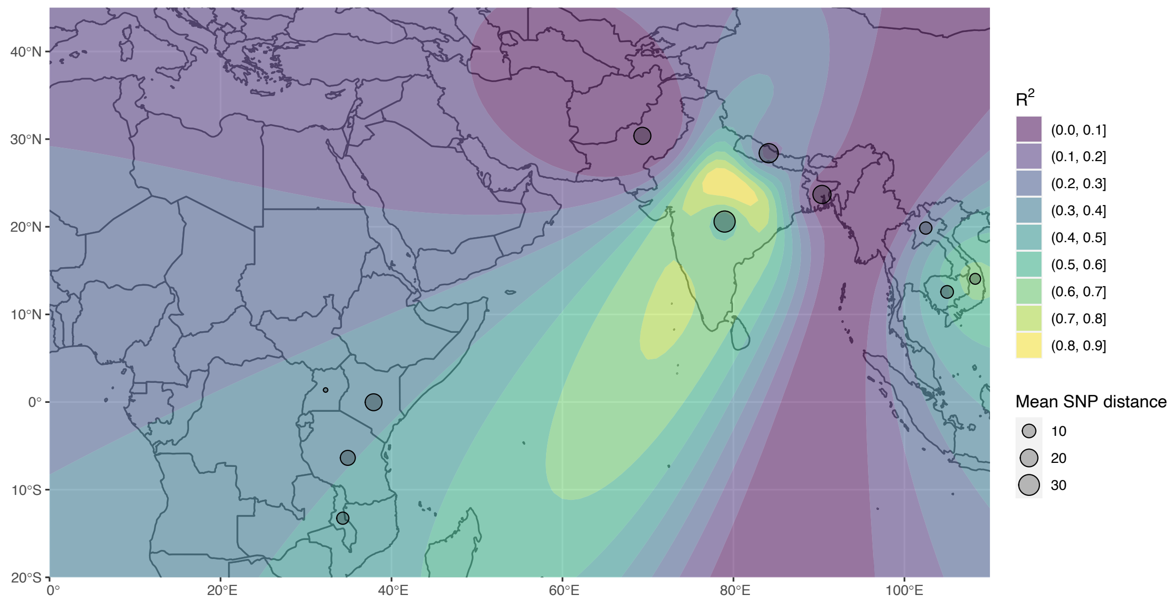

(**b**) Map of most likely origin of H58 *S*. Typhi. Grey circles represent the geographic locations of samples included in the analysis. Circles sizes are proportional to within-population genetic diversity. The heatmap represents the represents the R^2 of the log-linear model relating geographic distance and genetic diversity considering the origin at that geographic point, with light shades representing more likely origins (higher R^2).

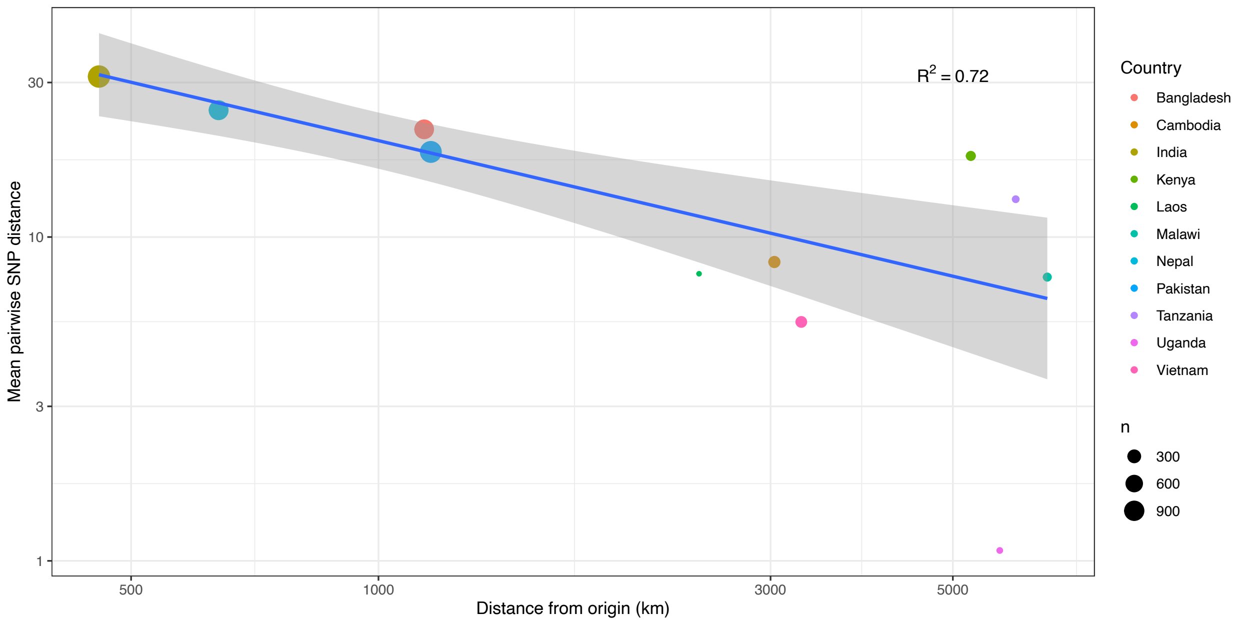

Supplementary Figure 7. Genetic diversity and distance from the geographic origin of H58 *S.* Typhi including Asian and African isolates. (a) Correlation between geographic distance (log km^2) and genetic diversity (log mean pairwise distance) for the best supported origin. n represents the number of samples.

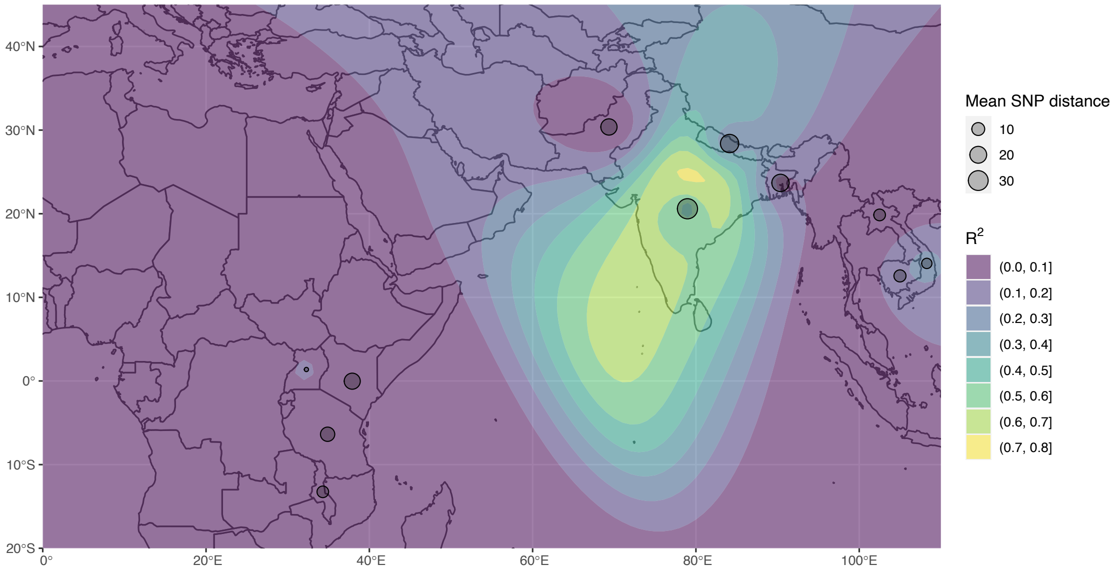

(b) Map of most likely origin of H58 *S*. Typhi. Grey circles represent the geographic locations of samples included in the analysis. Circles sizes are proportional to within-population genetic diversity. The heatmap represents the represents the R^2 of the log-linear model relating geographic distance and genetic diversity considering the origin at that geographic point, with light shades representing more likely origins (higher R^2).

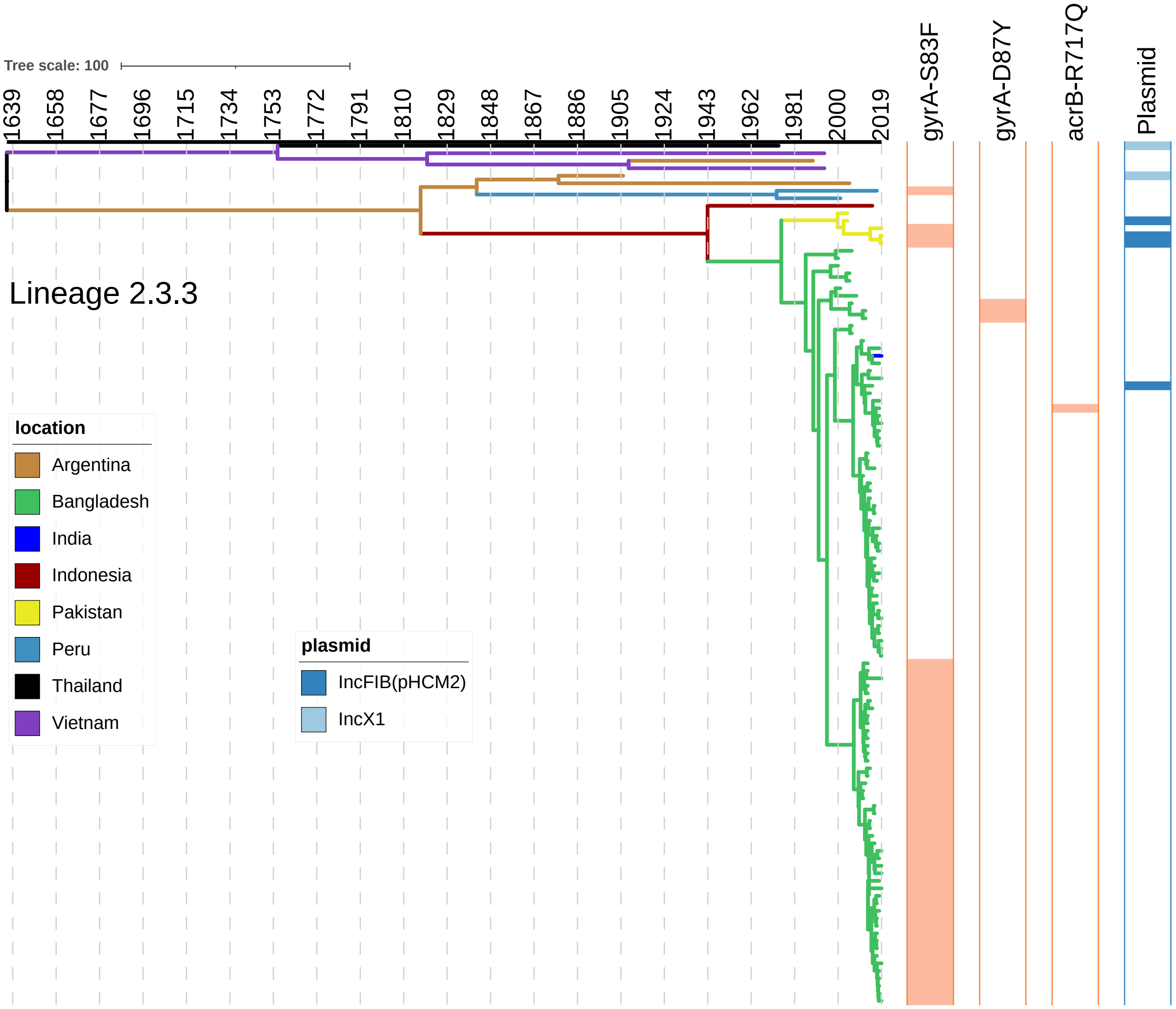

Supplementary Figure 8. Phylogeography and global expansion of *S.* Typhi lineage 2.3.3. **(a)** Maximum clade credibility tree (reconstructed using BEAST2) of genotype 2.3.3 *S.* Typhi isolates. The branches are in time scale in years and are colored according to the location of the most probable ancestor of descendant nodes. The scale bar indicates nucleotide substitutions per site.

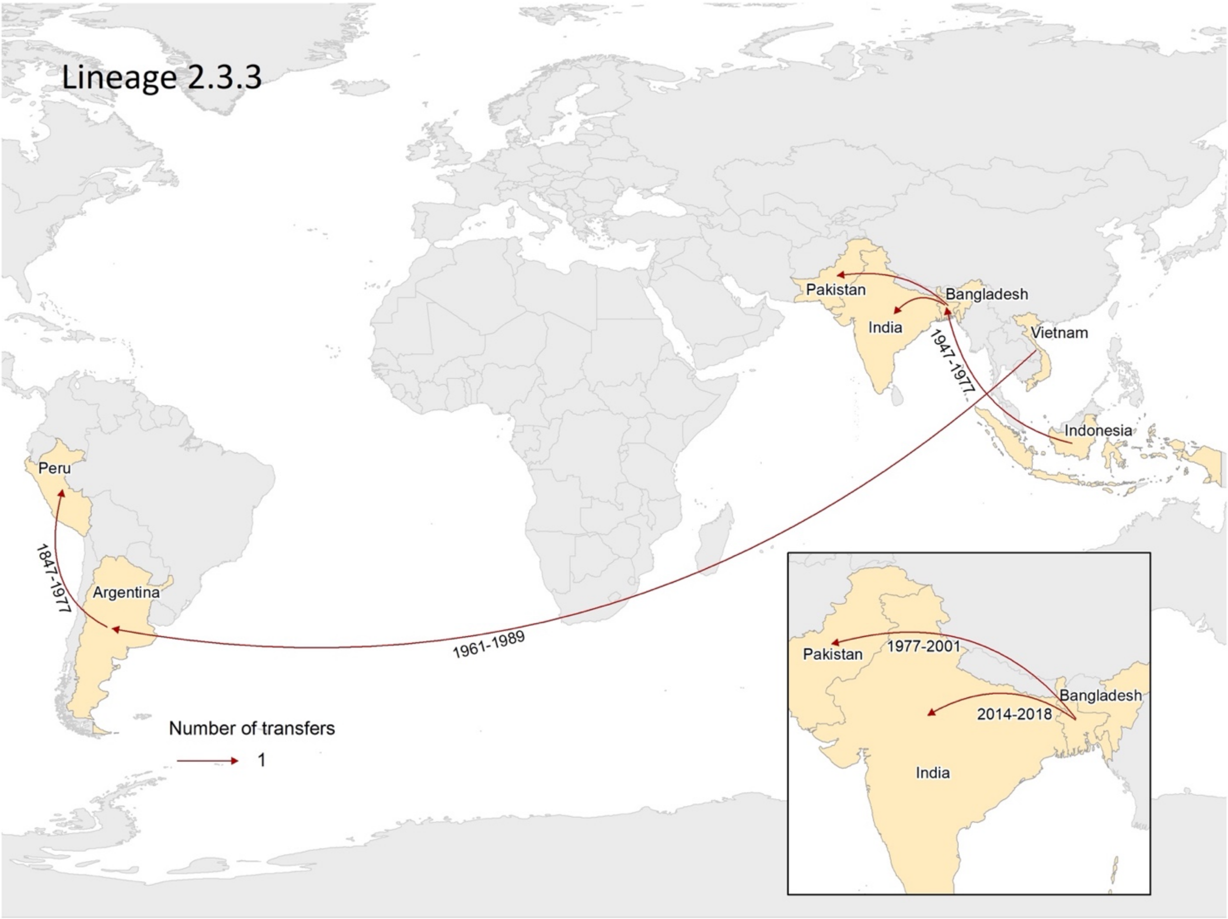

(b) Geographical transfers within the 2.3.3 lineage, inferred from the phylogenetic tree. The size of each arrow indicates the relative number of likely transfers between the countries.

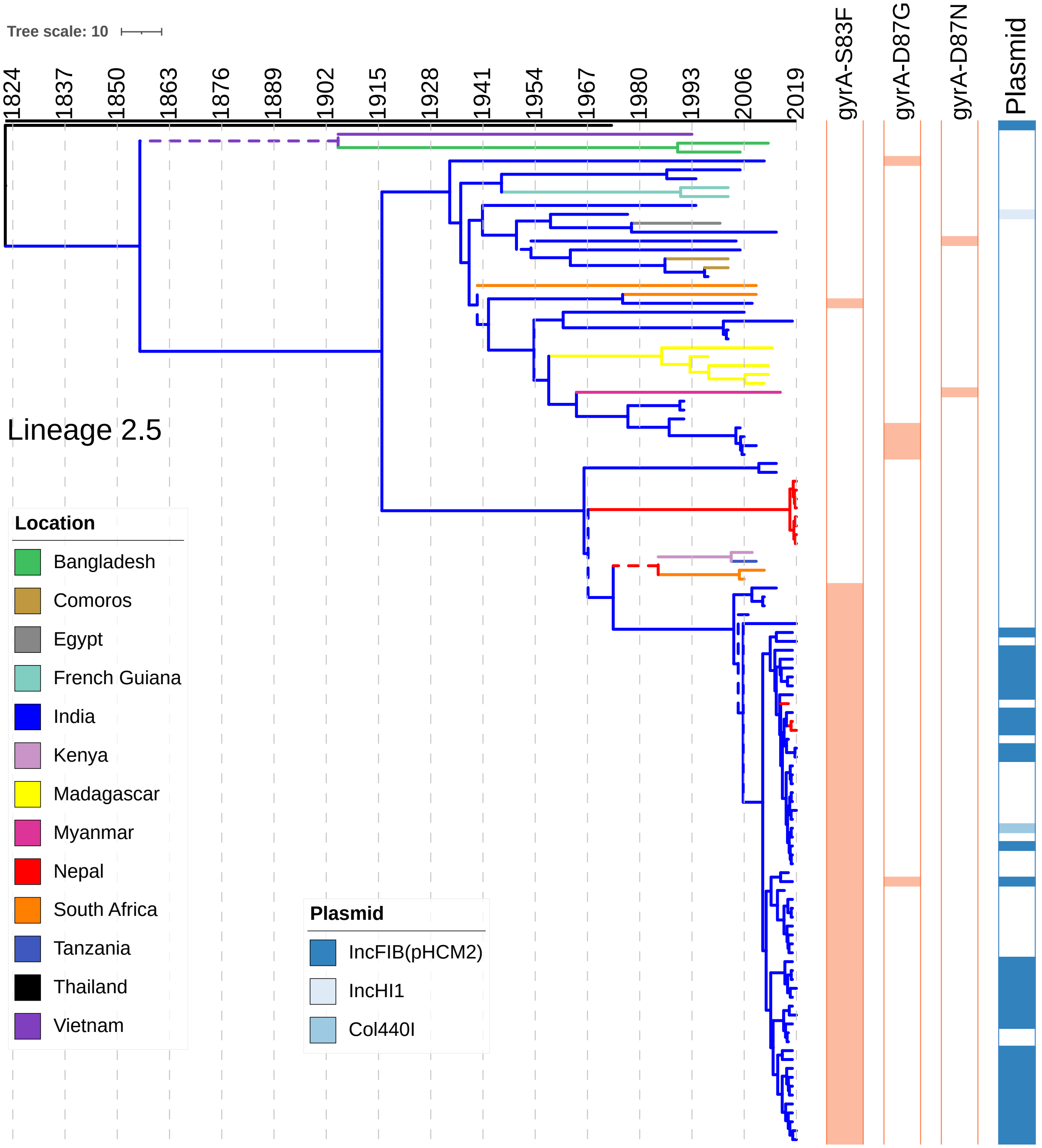

Supplementary Figure 9. Phylogeography and global expansion of *S.* Typhi lineage 2.5. **(a)** Maximum clade credibility tree (reconstructed using BEAST2) of genotype 2.5 *S.* Typhi isolates. The branches are in time scale in years and are colored according to the location of the most probable ancestor of descendant nodes. Branches are displayed as dashed lines when the posterior probability values were below 0.5. The scale bar indicates nucleotide substitutions per site.

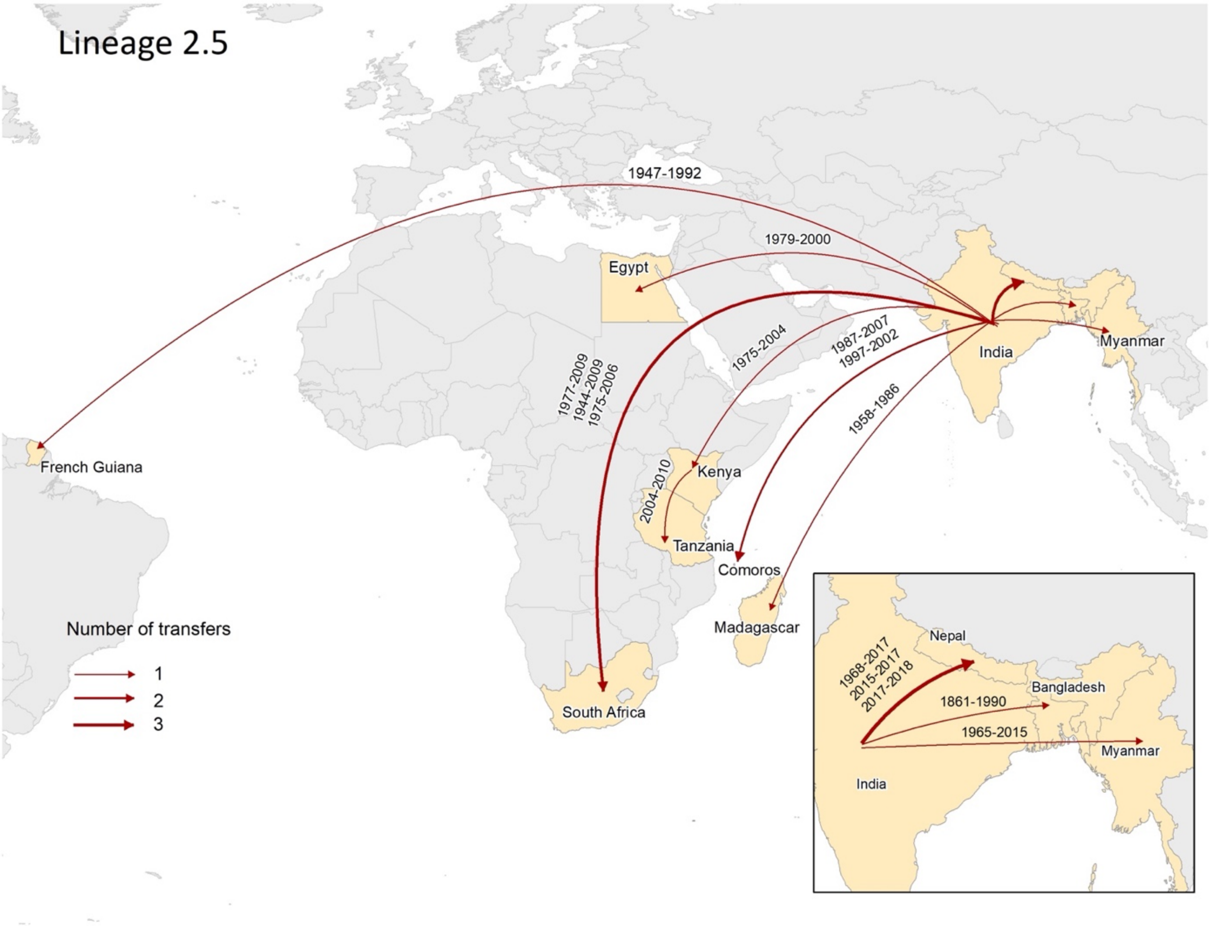

(b) Geographical transfers within the 2.5 lineage, inferred from the phylogenetic tree. The size of each arrow indicates the relative number of likely transfers between the countries.

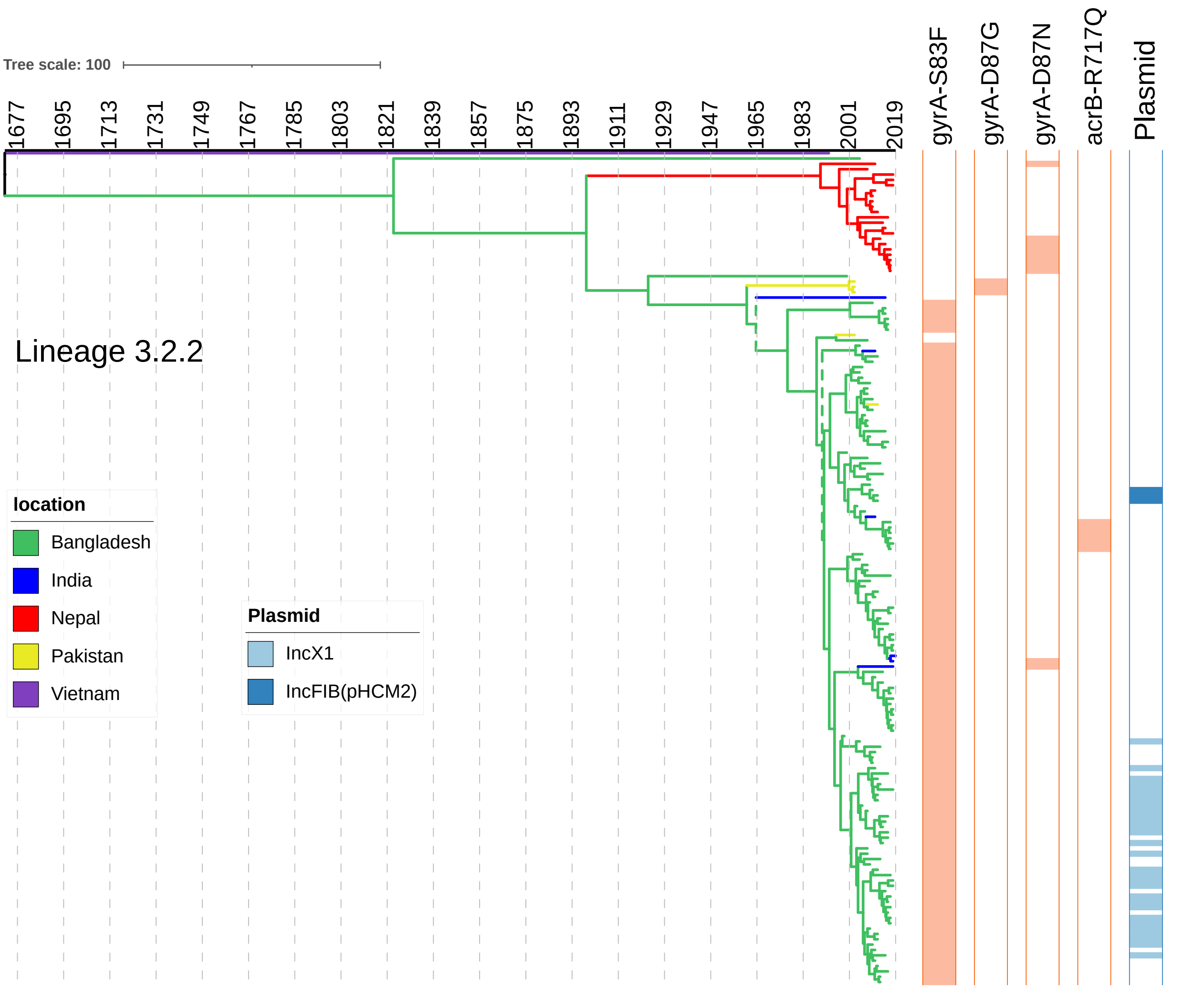

Supplementary Figure 10. Phylogeography and global expansion of lineage 3.2.2. **(a)** Maximum clade credibility tree (reconstructed using BEAST2) of genotype 3.2.2. *S.* Typhi isolates. The branches are in time scale in years and are colored according to the location of the most probable ancestor of descendant nodes. Branches are displayed as dashed lines when the posterior probability values were below 0.5. The scale bar indicates nucleotide substitutions per site.

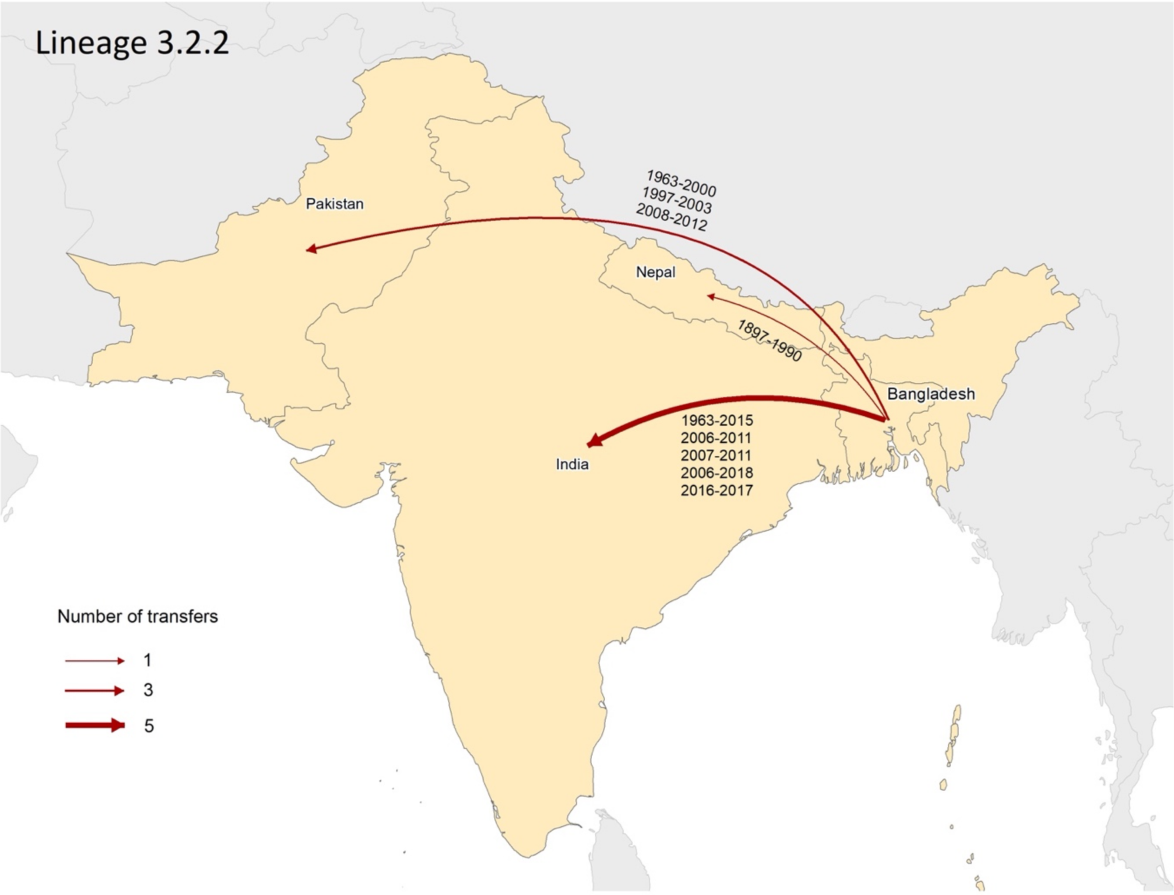

(b) Geographical transfers within the 3.2.2 lineage, inferred from the phylogenetic tree. The size of each arrow indicates the relative number of likely transfers between the countries.

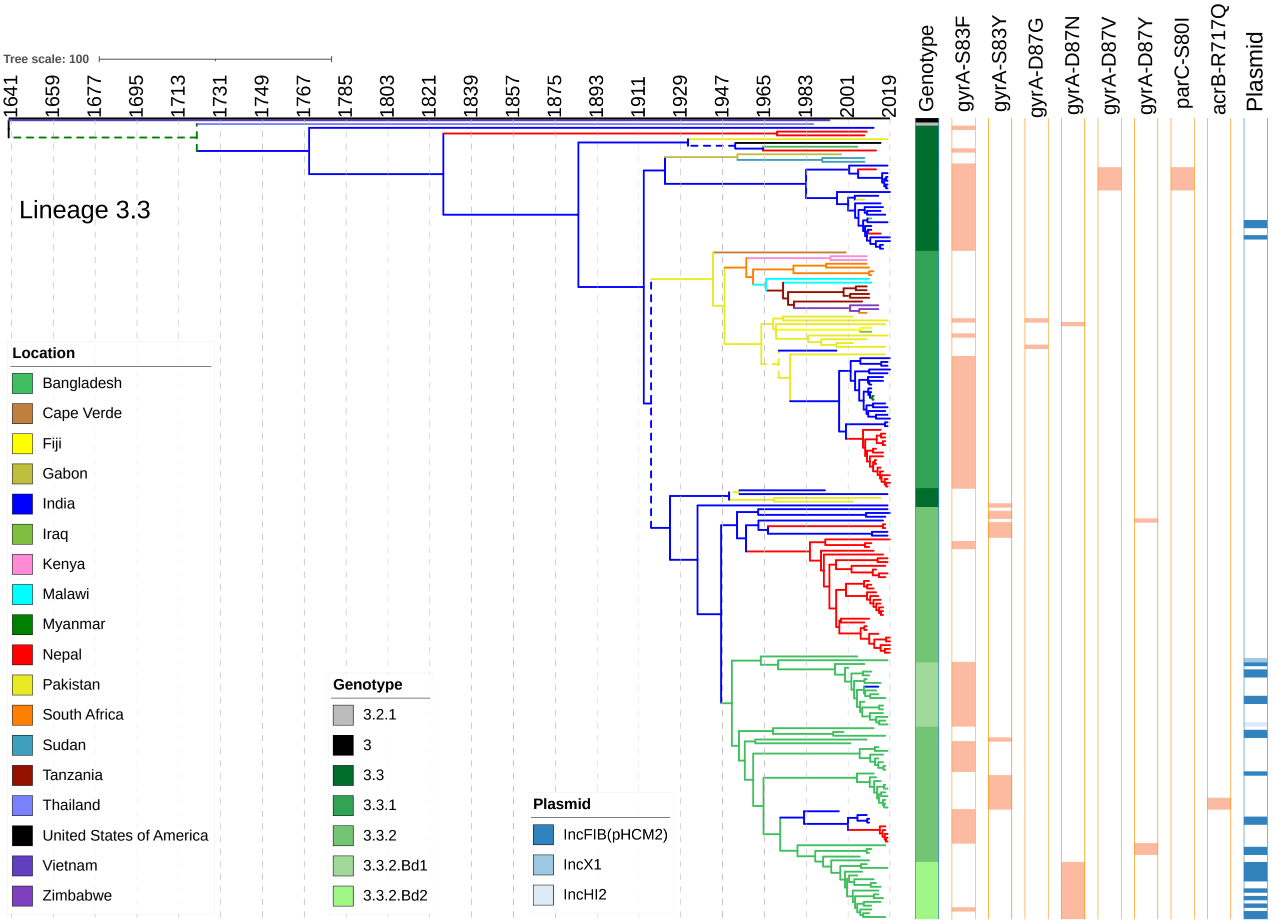

Supplementary Figure 11. Phylogeography and global expansion of *S.* Typhi lineage 3.3. **(a)** Maximum clade credibility tree (reconstructed using BEAST2) of genotype 3.3 *S*. Typhi isolates. The branches are in time scale in years and are colored according to the location of the most probable ancestor of descendant nodes. Branches are displayed as dashed lines when the posterior probability values were below 0.5. The scale bar indicates nucleotide substitutions per site.

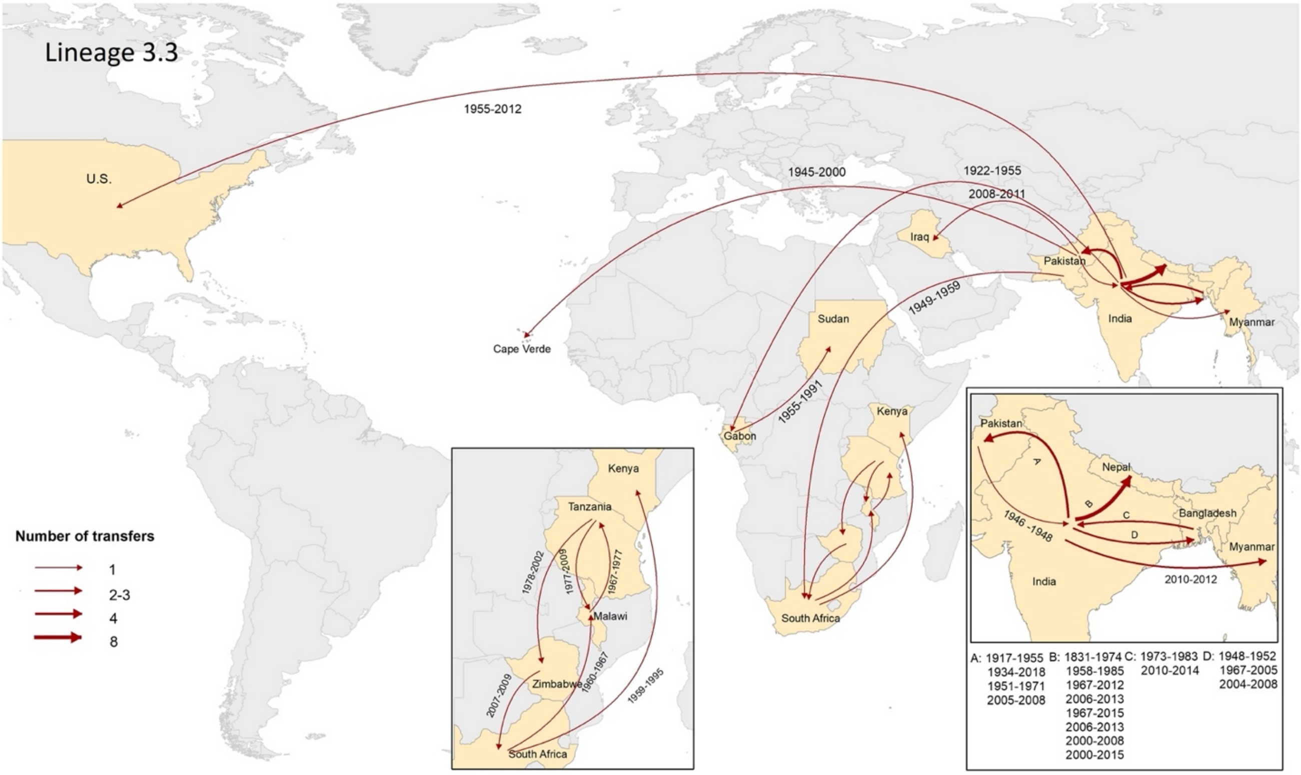

(b) Geographical transfers within the lineage 3.3 inferred from the phylogenetic tree. The size of each arrow indicates the relative number of likely transfers between the countries.

Supplementary Table 3. Temporal distribution of conferring-resistant genes involved with multidrug-resistant (MDR) genotype identified in *S*. Typhi isolates from our global collection.

**Genotype**	**No. of isolates and time range in years (%)**						
	**1951-1960**	**1961-1970**	**1971-1980**	**1981-1990**	**1991-2000**	**2001-2010**	**2011-2020**
***bla*$_{\textbf{TEM-1}}$ + *catA1* + *dfrA7* + *sul1* and/or *sul2*[a]**			1 (5)	4 (6.5)	173 (39.1)	420 (29.1)	1,449 (25.8)
bla$_{\text{TEM-1}}$ + *catA1* + *dfrA7*						1 (0.1)	2 (0.05)
bla$_{\text{TEM-1}}$ + *catA1* + *sul1* and/or *sul2*						17 (1.2)	35 (0.6)
bla$_{\text{TEM-1}}$ + *dfrA7* + *sul1* and/or *sul2*	1 (14.3)				2 (0.5)	23 (1.6)	17 (0.3)
bla$_{\text{TEM-1}}$ + *sul1* and/or *sul2*	1 (14.3)					48 (3.3)	100 (1.8)
bla$_{\text{TEM-1}}$					5 (1.1)		4 (0.08)
catA1 + *dfrA7*+ *sul1* and/or *sul2*					5 (1.1)	36 (2.5)	57 (1)
catA1 + *sul1* and/or *sul2*			1 (5)	4 (6.5)	5 (1.1)	3 (0.2)	11 (0.2)
catA1						2 (0.2)	1 (0.03)
dfrA7 + *sul1* and/or *sul2*							12 (0.2)
sul1 and/or *sul2*							2 (0.05)
none	5 (71.4)	1 (100)	18 (90)	54 (87)	252 (57.1)	893 (61.8)	3,929 (69.9)
Total	7 (100)	1 (100)	20 (100)	62 (100)	442 (100)	1,444 (100)	5,619 (100)
**MDR location (genes)[a]**							
Chromosome				4 (100)	34 (19.7)	218 (51.9)	1,264 (87.2)
Plasmid (IncHI1)			1 (100)		139 (80.3)	202 (48.1)	185 (12.8)
Total			1 (100)	4 (100)	173 (100)	420 (100)	1449 (100)

We classified isolates as multidrug-resistant (MDR) if they simultaneously contained genes conferring resistance to ampicillin (*bla*$_{\text{TEM-1}}$), chloramphenicol (*catA1*), and trimethoprim-sulfamethoxazole (*dfrA7* plus [*sul1* and/or *sul2*]).

Supplementary Table 4. Frequency of QRDR mutation patterns present among 7,658 *S.* Typhi isolates from the global collection.

**Mutation profile**		**Number of isolates n = 5613 (%)**
**Single mutant**		
	gyrA-S83F	3950 (70.39)
	gyrA-S83Y	927 (16.52)
	gyrA-D87N	126 (2.24)
	gyrA-D87G	32 (0.57)
	gyrA-D87Y	17 (0.30)
	parC-S80I	4 (0.07)
**Total**		5057 (90.09)
**Double mutant**		
	gyrA-S83F, parC-S80I	57 (1.02)
	gyrA-S83F, parC-E84G	38 (0.68)
	gyrA-S83F, parC-E84K	13 (0.23)
	gyrA-D87N, gyrA-S83F	4 (0.07)
	gyrA-S83Y, parC-S80R	4 (0.07)
	gyrA-D87G, gyrA-S83F	2 (0.04)
	gyrA-S83F, parC-S80R	1 (0.02)
**Total**		119 (2.12)
**Triple mutant**		
	gyrA-D87N, gyrA-S83F, parC-S80I	408 (7.27)
	gyrA-D87V, gyrA-S83F, parC-S80I	15 (0.27)
	gyrA-D87G, gyrA-S83F, parC-E84K	8 (0.14)
	gyrA-D87G, gyrA-S83F, parC-E84G	1 (0.02)
	gyrA-D87N, gyrA-S83F, parC-E84G	1 (0.02)
	gyrA-D87N, gyrA-S83F, parC-E84K	1 (0.02)
	gyrA-D87G, gyrA-S83F, parC-S80I	1 (0.02)
	gyrA-D87G, gyrA-S83Y, parC-S80I	1 (0.02)
	gyrA-D87N, gyrA-S83F, parC-S80R	1 (0.02)
**Total**		437 (7.79)

Supplementary Table 5. Comparison between genotypic and phenotypic resistance profile among S. Typhi isolates from our collection.

Antimicrobial resistance genotype		No. of isolates with indicated phenotype			
	No.	Susceptible	Intermediate	Resistant	N/A
**Ampicillin**					
*bla*TEM-1	474	18	3	442	11
**Co-trimoxazole**					
dfrA7 + *sul1* + *sul2*	850	26	7	806	11
dfrA7 + *sul1* only	55	10	2	41	2
dfrA7 + *sul2* only	2	-	-	2	-
sul1 + *sul2*	2	2	-	-	-
sul2	69	68	-	1	-
**Chloramphenicol**					
catA1	902	36	6	845	15
**Ceftriaxone**					
*bla*CTX-M-15	465	11	1	446	7
**Ciprofloxacin**					
1 QRDR mutation	2,915	93	2,040	737	45
2 QRDR mutation	64	1	28	35	-
3 QRDR mutation	286	2	11	272	1
qnrS	518	7	15	488	8
**Azithromycin**					
acrB-R717L	3	-	-	3	-
acrB-R717Q	33	9	-	22	2

Supplementary table 6. Estimated times of the most recent common ancestors (tMRCAs) of the main clades, with calendar years and most probable locations, from discrete phylogeography of the *S*. Typhi isolates.

Genotype	tMCRA	Country	Substitution rate
**2.3.3**	1817	Vietnam	1.55 x 10^{-7}
**2.5**	1856	India	2.85 × 10^{-7}
**3.2.2**	1899	Bangladesh	9.81 x 10^{-8}
**3.3**	1770	India	1.28 × 10^{-7}
**4.3.1**	1984	India	7.98 × 10^{-6}
